# Nanoscale Optical Inhomogeneities From Compositional Segregation Within Individual GaN‐on‐Si Quantum Wells

**DOI:** 10.1002/advs.76414

**Published:** 2026-07-06

**Authors:** Jing‐Yang Chung, Tara P. Mishra, Zackaria Mahfoud, Thomas E. Gage, Jianguo Wen, Katherine P. Rice, Isabelle Martin, Li Zhang, Govindo J. Syaranamual, Stephen J. Pennycook, Silvija Gradečak, Pieremanuele Canepa, Michel Bosman

**Affiliations:** ^1^ Department of Materials Science and Engineering National University of Singapore Singapore Singapore; ^2^ Singapore‐MIT Alliance for Research and Technology Singapore Singapore; ^3^ Institute of Materials Research and Engineering, Agency for Science Technology and Research (A*STAR) Singapore Singapore; ^4^ Center For Nanoscale Materials Argonne National Laboratory Lemont Illinois USA; ^5^ CAMECA Instruments, Inc. Madison Wisconsin USA; ^6^ Department of Electrical and Computer Engineering University of Houston Houston Texas USA; ^7^ Texas Center for Superconductivity University of Houston Houston Texas USA

**Keywords:** atom probe tomography, cathodoluminescence, defects, GaN, LED, optical inhomogeneities, phase separation

## Abstract

Modern high‐electron‐mobility transistors (HEMTs) and light‐emitting diodes (LEDs) are engineered around epitaxial indium gallium nitride (In_x_Ga_1 − x_N) heterostructures. We apply the highest‐resolution structural, elemental, and optical characterization to a series of systematically fabricated GaN‐on‐silicon (Si) epitaxial heterostructures and demonstrate hitherto unresolved nanoscale optical inhomogeneities in individual In_x_Ga_1 − x_N quantum wells. Direct correlation of atom probe tomography and scanning transmission electron microscopy with cathodoluminescence collectively confirms that these optical inhomogeneities result from compositional segregation that only appears in high indium‐content In_x_Ga_1−x_N specimens, but not in low indium‐content quantum wells, thereby elucidating the origin of injection‐current‐induced blueshifts in long wavelength LEDs. Density functional theory (DFT) calculations of the various alloy mixing energies indicate that the relaxation of the epitaxial in‐plane strain stabilizes specific In_x_Ga_1−x_N compositions. Our findings suggest that proper use of strain management and careful selection of alloy compositions are necessary to control local phase separation. This work presents a path to high‐mobility strain engineering in HEMTs and homogeneous, long‐wavelength light emission in LEDs.

## Graphical abstract

This work visualizes local variations in luminescence and relates them to nanoscale chemical inhomogeneities in single In_x_Ga_1−x_N quantum wells. We demonstrate that the elemental segregation is only inherent to quantum wells with high indium content. Density functional theory shows this to be the result of strain‐induced phase stabilization, explaining why an increased injection current causes blue‐shifted emission(s) for long wavelength GaN‐based LEDs.

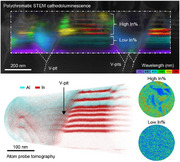

## 1 Introduction

Group‐III nitride binary compounds and their alloys are direct bandgap semiconductors spanning an extensive range of energies, from 0.7 eV (indium nitride, InN) to 6.04 eV (aluminum nitride, AlN), offering the potential of bandgap engineering from near infrared to deep ultraviolet [1]. This flexible tuning property has made them among the most widely used materials for low‐cost, high‐efficiency lighting and micro‐display sources [2, 3, 4], as well as high‐electron‐mobility transistors (HEMTs) [5, 6]. In particular, upcoming applications, such as virtual and augmented reality (AR/VR), require full color displays with small pixel pitches [7], necessitating the use of a single material without color down‐conversion. III‐nitride‐based heteroepitaxial films, with their full tunability across the visible spectrum, may therefore be the most suitable solid‐state light‐emitting material by choice.

**FIGURE 1:**
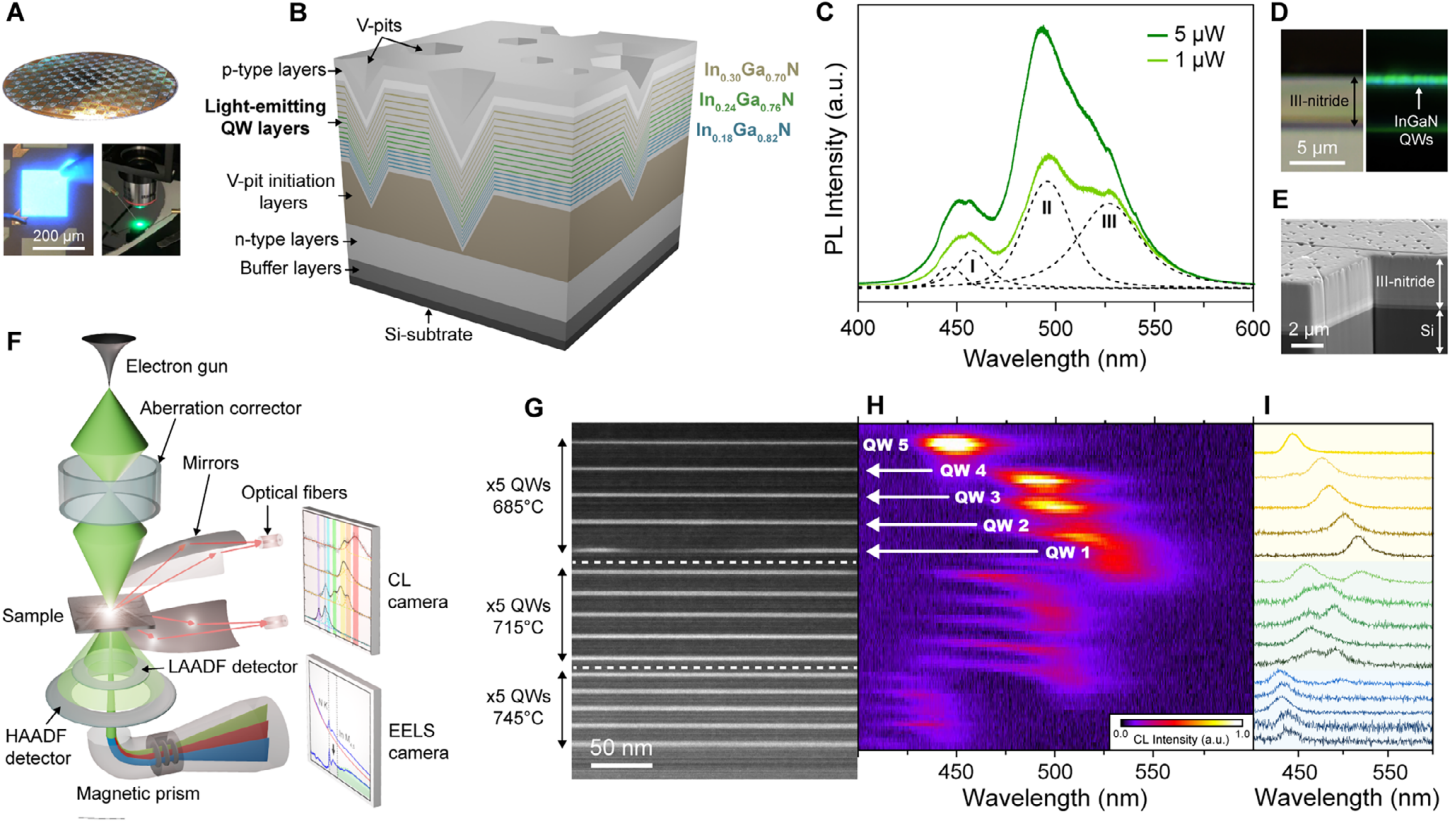
GaN‐on‐Si heterostructures. (A) One of our 8” wafers with In_x_Ga_1 − x_N heterostructures grown on a silicon substrate. Two example *p–n* junctions on this wafer are shown: one that locally emits blue light (bottom left), and another one emitting green light (bottom right). (B) One of the fabricated GaN‐on‐Si heterostructures, with three different sets of In_x_Ga_1 − x_N QWs, grown with decreasing temperatures of 745°C (blue), 715°C (green), and 685°C (yellow). (C) Photoluminescence spectra acquired from the heterostructure in (B). Dotted lines show Voigt fits for the spectrum acquired at 1 µW. (D) Dark field light microscope image (left) and fluorescence light microscopy image (right) of a cross‐sectional sample, showing green–blue emission from the QWs. (E) Scanning electron microscopy secondary electron image showing the surface and cross‐section of the same heterostructure, and at roughly the same scale. (F) Schematic representation of our set‐up for atomic‐resolution imaging, elemental characterization, and nano‐optical spectroscopy using STEM‐based EELS and cathodoluminescence (CL) with a focused electron beam. (G) Cross‐sectional STEM HAADF image of the structure presented in panels (B–E), showing the 15 In_x_Ga_1 − x_N QWs deposited at 745°C, 715°C, and 685°C, respectively. (H) CL spectral profile integrated across the 15 QWs as a function of the emission wavelength and position. (I) Extracted CL spectra from each QW.

Visible light is generated from these devices by the formation of a *p–n* heterojunction. Thin layers of the alloy In_x_Ga_1−x_N are epitaxially grown between GaN layers of a larger bandgap energy (Figure 1A,B), and the creation of this double heterojunction is referred to as a quantum well (QW). Under forward bias, minority carriers radiatively recombine with majority carriers at the heterojunctions, resulting in the emission of photons with energy close to the bandgap of the InGaN material. However, even slight variations of QW dimensions, chemical compositions, and/or strain can lead to deviations from the desired light‐emitting diode (LED) emission wavelength [8]. Atomic‐scale crystal defects commonly nucleate at the indium‐doped QWs and have been found to alter luminescence properties [9, 10, 11, 12, 13]. Larger defects, such as V‐pits (Figure 1B), which are three‐dimensional (3D) inverted hexagonal pits that nucleate from threading dislocations [14, 15, 16], likewise result in additional radiative recombination paths. These factors, together with the increasing quantum confined Stark effect, lead to a decrease in the external quantum efficiencies in In_x_Ga_1 − x_N LEDs from over 80% and 50% for blue and green emission [17, 18], to under 35% and 10% for yellow and red emission [19, 20], respectively.

However, it is not straightforward to gain direct insight into the relationship between atomic‐scale structural defects and the light emission properties of these QW devices. While optical techniques, such as fluorescence [21], photoluminescence (PL) mapping [22], and near‐field scanning optical microscopy [23], have been used for screening the optical properties of GaN surface structures, they provide insufficient spatial resolution to establish a direct link with atomic‐scale structural or compositional defects. For example, Figure 1C displays representative PL spectra of the device architecture depicted in Figure 1B. This is a GaN‐on‐Si heterostructure with three sets of QWs of chemical compositions typically used in In_x_Ga_1 − x_N LEDs and HEMTs. Fluorescence light microscopy on this device in the cross‐section is shown in Figure 1D. Green–blue emission can be seen from a band that is almost a micrometer thick, without being able to resolve the closely spaced individual QWs. A 3D image of this structure is also visible in the scanning electron microscope (SEM) image of Figure 1E, captured at roughly the same scale as the fluorescence image. Based only on the PL spectra and knowing that three series of QWs are present in the sample, it is reasonable to assume that the three spectral peaks (labelled as I, II, and III in Figure 1C) originate from these QWs (labelled with blue, green, and yellow in Figure 1B). However, our nano‐optical measurements will show that this assumption is not necessarily valid.

Cathodoluminescence (CL) spectroscopy bypasses the fundamental resolution limit of light microscopy. This is achieved by using the focused electron beam of a scanning transmission electron microscope (STEM), as schematically shown in Figure 1F, to excite the optical transitions locally with nanometer spatial precision [24]. The sample subsequently emits in the far‐field, and this emission is measured with the help of ellipsoidal or parabolic mirrors, optical fibers, and an appropriate detector. STEM‐based CL has been used for resolving the emission of individual QWs [25, 26], quantum disks [27, 28], optical bound‐states‐in‐the‐continuum [29], emission inhomogeneities [30, 31, 32, 33, 34], and has provided insight on the optical properties of extended defects [11, 12, 13]. However, this technique has not yet been used to study the luminescence centers in long‐wavelength LED architectures. STEM CL can also be coupled with other high‐resolution imaging and spectroscopic analysis in the same microscope, allowing direct structure‐property understanding down to the atomic scale [35]. These techniques include high‐angle annular dark‐field (HAADF) for atomic mass‐contrast imaging, low‐angle annular dark‐field (LAADF) for strain‐contrast imaging, and electron energy loss spectroscopy (EELS) for elemental and bonding information, as was discussed in a recent review [36].

Figure 1G shows a HAADF image of the 15 light‐emitting QWs separated into three sets, grown at 745°C, 715°C, and 685°C, respectively, with the CL spectral profile of each QW plotted in Figure 1H. Despite the same deposition temperature of 685°C, it becomes apparent through CL that only the bottom‐most QW emits at ∼525 nm, whereas the remaining QWs in the 685°C set have blue‐shifted up to 450 nm for the uppermost QW. This might be due to a depletion in the In‐content in the upper QWs, otherwise known as the compositional pulling effect [37], and is supported by the energy dispersive x‐ray spectroscopy (EDS) results shown in Figure S4. Another unexpected observation is the double emission peak for the QWs deposited at 715°C, shown in Figure 1I. No extended defect nucleates in these QWs, so this STEM CL observation may allude to compositional segregation within individual QWs. This example illustrates the versatility of STEM CL in providing insight into local luminescence that surpasses the capabilities of PL measurements.

In this work, we demonstrate the origins of emission inhomogeneities in In_x_Ga_1 − x_N quantum wells by combining systematic epitaxial growth, ultrahigh‐resolution imaging and spectroscopy, as well as DFT calculations. The insight that is gained will allow a deeper understanding of the origin of the commonly observed inhomogeneous spectral emission in long‐wavelength In_x_Ga_1 − x_N LEDs. By refining the analysis to increasingly smaller volumes, we concluded that the source of this effect is within individual QWs, requiring a combination of very high‐resolution techniques. We previously demonstrated that electron spectroscopy is not ideally suited for measuring local elemental fluctuations in QWs because fluctuations only a few nanometers in size average out over the approximately 50 nm thickness of TEM samples [38]. Instead, an inherently 3D nanoscale characterization technique is needed that can distinguish the compositional inhomogeneity at this length scale [39]. For this purpose, we utilize atom probe tomography (APT) to directly correlate the local optical emission with the 3D chemical structure. We are not the first ones to apply APT to In_x_Ga_1−x_N films [40]; Tang et al. and Humphreys et al. have previously used APT to reveal compositional fluctuations in non‐polar In_x_Ga_1 − x_N QWs grown in the *a*‐plane, contrasted to the more uniform elemental distributions in polar QWs grown in the *c*‐plane [41, 42]. Dimkou et al. previously performed in situ PL during the APT acquisition on blue‐emitting In_x_Ga_1 − x_N quantum dot (QD) LEDs [43], thereby allowing the final QD layer to be optically resolved. Our correlative STEM CL and APT approach establishes a previously inaccessible visual link between nanoscale emission variations in individual QWs and their local elemental distribution and growth conditions. Understanding these vital relations will then permit the targeted engineering of more efficient QWs for future optoelectronics and HEMTs, negating the need for extensive “trial and error” in the device fabrication process.

## 2 Results and Discussion

**FIGURE 2:**
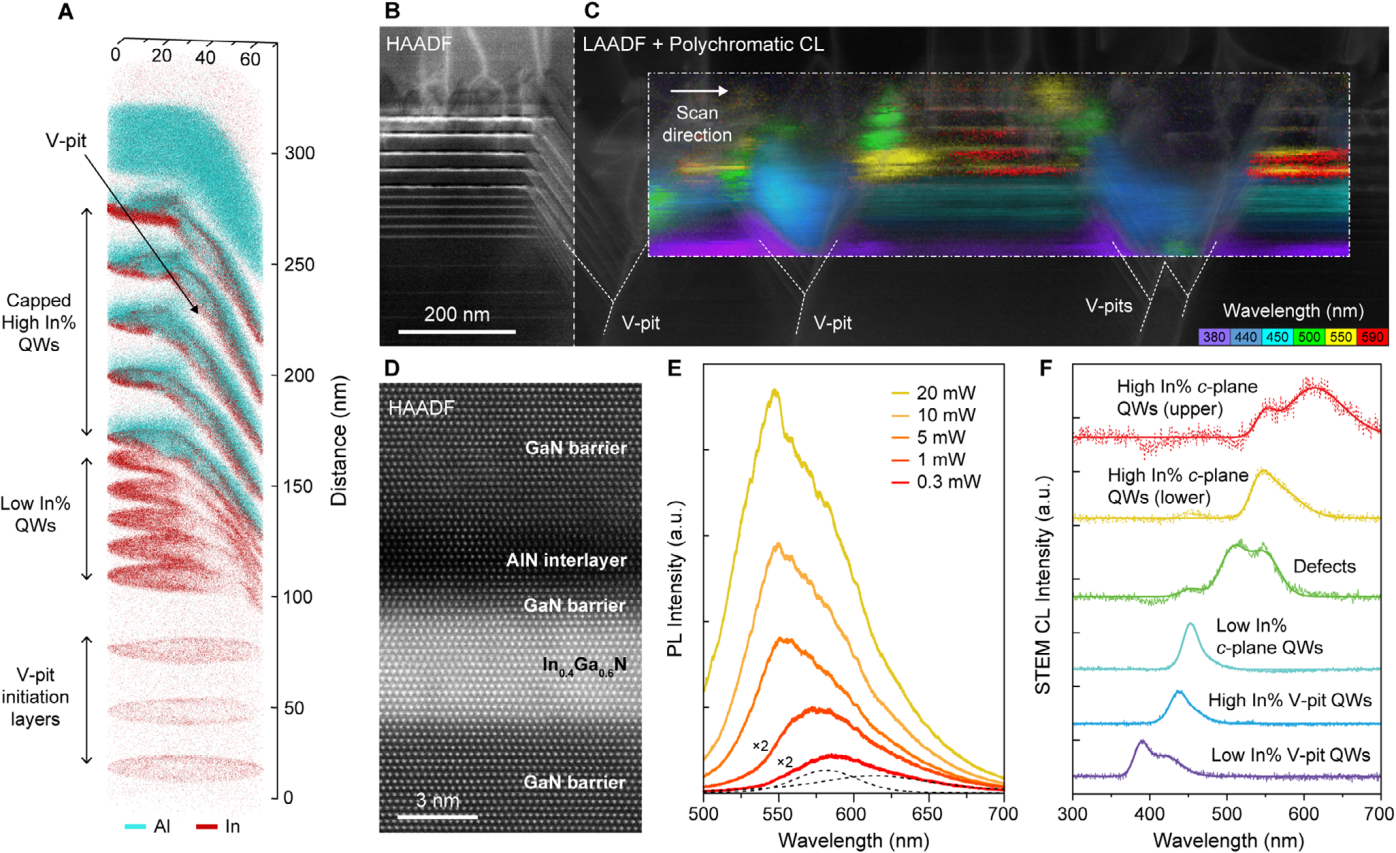
Nanoscale optical inhomogeneities in GaN‐on‐Si QWs. (A) 3D atom probe tomography reconstruction of an amber‐red emitting In_x_Ga_1 − x_N heterostructure comprising five sets of high In‐content QWs capped with AlN strain‐compensating layers, above five sets of low In‐content QWs. (B) Cross‐section STEM HAADF image, and (C) adjoining LAADF image with the spatially resolved polychromatic CL map overlaid onto the dashed‐boxed region. This image set was acquired in the [$11\bar{2}0$] orientation. (D) Atomically resolved HAADF image of a single In_x_Ga_1 − x_N/GaN/AlN/GaN stack. (E) Power‐dependent PL spectra measured on this sample. (F) CL local emission spectra of the various light‐emitting features.

We fabricated a blue‐ and red‐emitting structure with five high In‐content, red‐emitting In_x_Ga_1 − x_N QWs deposited at 630°C, above five low In‐content, blue‐emitting In_x_Ga_1 − x_N QWs deposited at 760°C. The high‐In QWs are capped with AlN strain‐compensating interlayers, a strategy we borrowed from recent work on high‐efficiency red LEDs architectures [20, 21, 44, 45, 46, 47, 48]. These thin interlayers have been shown to prevent indium desorption during growth, and reduce operating voltages by suppressing electron overflow while allowing tunnelling‐assisted hole injection [18]. This structure is visualized via the 3D APT data presented in Figure 2A, in which indium atoms are colored red and aluminum atoms cyan. The left half of the measured volume shows the flat *c*‐plane QWs, while the right half was measured in a V‐pit. The V‐pit density is relatively high in this sample, similar to the threading dislocations density: 2 × 10^9^ cm^−2^. An equivalent sample location is shown in Figure 2B, with atomic‐resolution detail for one of the single AlN‐capped QWs given in Figure 2D. An overview of this QW structure is presented in Figure S1, along with the Supplementary Video for a rotated view.

Power‐dependent PL measurements (Figure 2E) reveal three primary emissions: at low laser power (<1 mW), the 580 and 625 nm peaks are roughly equal in intensity; however, the latter rapidly becomes saturated at higher laser powers. Above 5 mW, the higher energy emission at ∼550 nm emerges and begins to dominate. Other studies on red In_x_Ga_1 − x_N LEDs have also reported the appearances of secondary blueshifted emission peaks [20, 48, 49, 50], or lopsided spectra [44, 51, 52, 53], when the injection current was increased. The ability to measure luminescence at the nanoscale makes STEM‐based CL a unique method for determining the source of this emission.

In Figure 2C, the LAADF image continues horizontally from the HAADF image in Figure 2B and is overlaid with a measured hyperspectral CL map in the boxed region. This map combines individual CL maps from the indicated wavelengths (see Figure S5 for the individual maps), revealing the exact positions of the various luminescence centers. Local emission spectra from the multiple features from this area are plotted in Figure 2F. Further examples of these same optical inhomogeneities are also shown through CL maps and point spectra in Figures S6 and S7, which were acquired across multiple TEM samples with different zone axes, varying STEM CL setups, and scanning directions perpendicular to the QWs. A few observations can be made directly: as expected, the emission redshifts from low to high indium content, both for the *c*‐plane (horizontal) QWs and for the V‐pit sidewall (diagonal) QWs.

In contrast, when comparing the same In‐content QWs in the *c*‐planes with those on the sidewalls of the V‐pits, a blueshift is observed. For the high indium QWs, an emission shift from 550 to 440 nm is observed from the yellow to the blue plot in Figure 2F, while the low In% QWs shift from 450 to 380 nm. This is likely the result of reduced indium and enhanced quantum confinement at the sidewall QWs. A large‐area example of this effect from the low In% QWs is shown in Figure S8.

Through STEM CL, we observe that the AlN‐capped high In‐content QWs emit at 590 nm, but surprisingly, also at ∼550 nm. Further point spectra taken at the high In‐content QWs suggest that this broad peak can be separated into two components at 580 and 625 nm, as shown in Figure S5. Together, these two signatures closely match the three emission peaks measured with PL. Previous studies have attempted to understand optical inhomogeneity in red LEDs architectures. Samuel et al. reported separate emission peaks at 588 and 610 nm in a long‐wavelength device [54] and speculated that these emissions arose from different QWs. Our significantly improved spatial resolution here reveals that such inhomogeneous emissions can *happen within individual* QWs.

**FIGURE 3:**
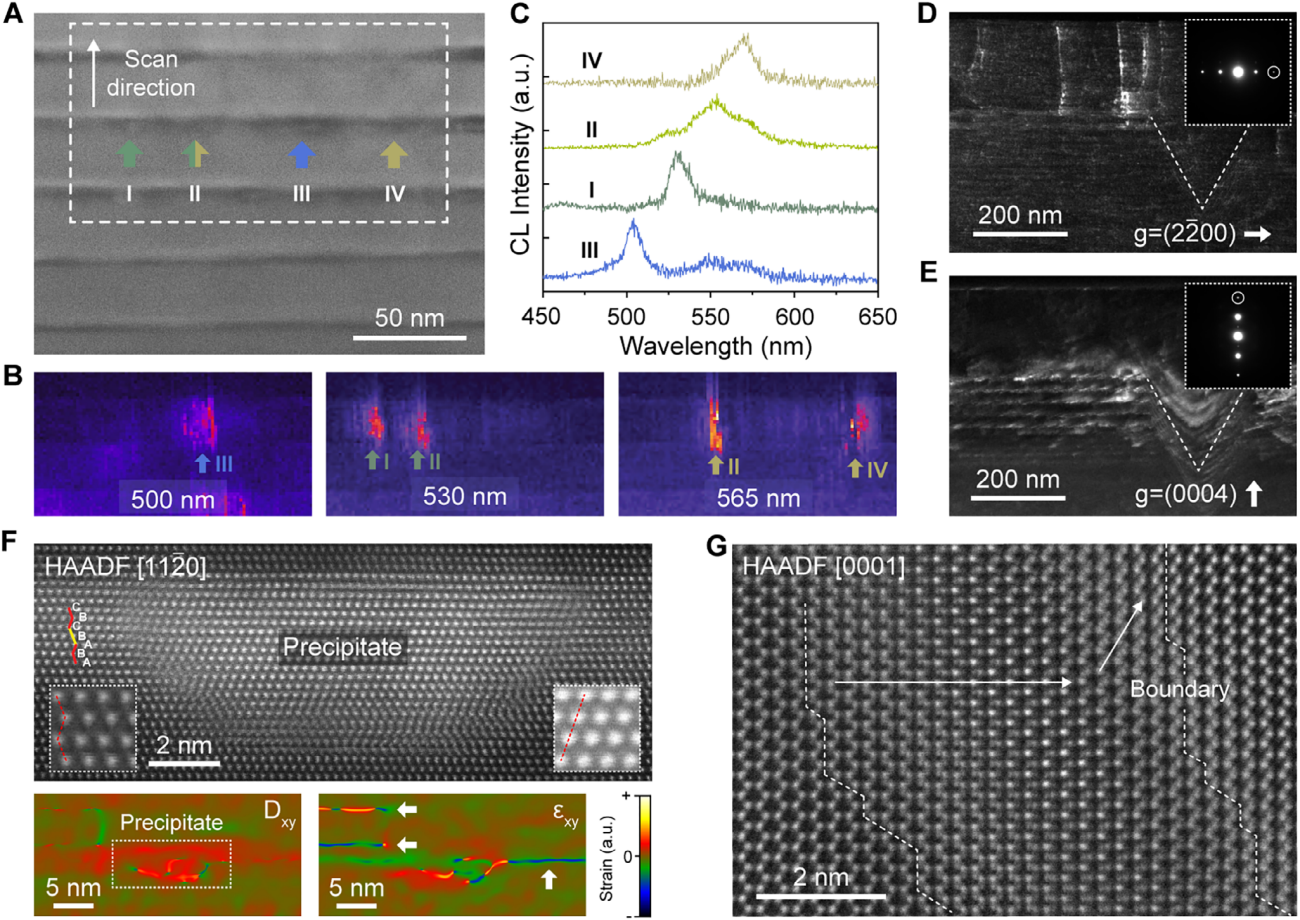
The crystal structure of luminescent defects. (A) Cross‐sectional STEM BF image of a region of In‐rich precipitates, and (B) corresponding monochromatic CL maps of the dashed boxed region at 500, 530, and 565 nm, with the individual spectra taken from the arrowed regions I–IV shown in (C). Cross‐sectional weak‐beam dark‐field TEM images of extended defects arising from the In‐rich precipitates from another, similar sample; taken with **g** vectors of (D) $\left(2\bar{2}00\right)$, and (E) (0004). The insets show the two‐beam conditions used for imaging. (F) Atomic HAADF image of an In‐rich precipitate in the [11$\bar{2}$0] zone‐axis, and larger field‐of‐view GPA dilation (*D_xy_*) and shear (*ε*_xy_) strain maps of the same region. (G) Atomic HAADF image of a defect boundary along the [0001] zone‐axis.

**FIGURE 4:**
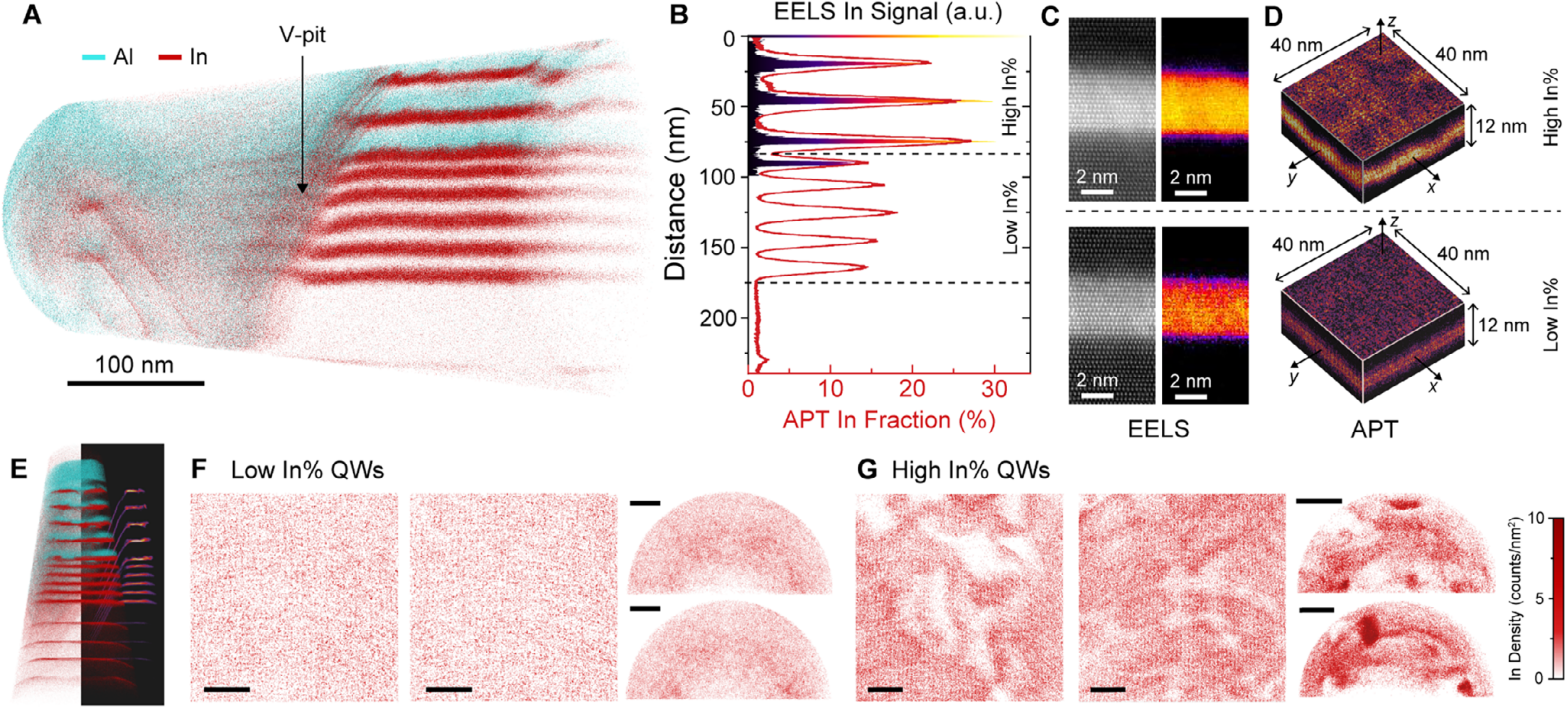
Nanoscale compositional inhomogeneities in GaN‐on‐Si QWs. (A) 3D APT reconstruction of the same red‐emitting InGaN heterostructure as presented in Figure 2. (B) Relative compositional line profile of indium calculated from both APT and STEM EELS, comparing the low and high In‐content QWs. The inset shows the HAADF and EELS map. (C) Higher magnification EELS maps of the high and low In‐content QWs (top and bottom, respectively). (D) APT projected density contour plots integrated in the *x*, *y*, and *z* directions of 40 × 40 × 12 nm slices extracted from the low and high In‐content QWs. (E) 3D APT reconstruction in the planar orientation and the projected indium density contour plot. (F,G) In‐plane projected APT indium density maps extracted from QWs in (A) and (E), showing 5 nm thick slices of individual QW, and binned 0.5 nm in the lateral direction. Scale bars are 20 nm.

The polychromatic map in Figure 2C also shows that the green to yellow emissions around 500–565 nm can arise from extended defects nucleated both in the vicinity of the V‐pits, and—as we will later show in Figure 3—within In‐rich precipitates in the *c*‐plane. The observation of these very localized crystal defect emissions may help to interpret previously reported inhomogeneous luminescence. These defects do not uniquely appear in red‐emitting structures, as shown by other studies that report similar V‐pit localized defects or In‐rich precipitates together with broad emission within this spectral range [16, 47]. Next, we will discuss these 500–565 nm defect emissions together with atomic‐resolved imaging in Figure 3, before discussing the 550–625 nm emission in Figure 4.

### 2.1 The Crystal Structure of Luminescent Defects

In our previous study [36], we examined the plasmon response of In‐rich precipitates using EELS mapping. We concluded that the precipitates are likely metallic In formed through the decomposition of non‐epitaxial InN. Here, in Figure 3A,B, we map the CL signal of these precipitates and unexpectedly observe luminescence at 500–565 nm (Figure 3C), similar to that observed from the V‐pit localized defects. Due to the metallic nature of the precipitates, radiative recombination is unlikely to arise within the precipitates themselves. However, further analysis (Figure 3D–F) demonstrates that additional extended crystal defects can nucleate alongside the precipitates. In Figure 3D,E, a weak‐beam dark‐field TEM set is shown, using the ***g*** vectors $\left(2\bar{2}00\right)$ and (0004). On the left of the V‐pit, vertical propagating strain contrast lines appear from the precipitates only in Figure 3D, which suggests that the defects have edge‐type character. For greater clarity, the equivalent STEM image of this region is shown in Figure S9, where both precipitates and defects are visible. This matches our atomic‐resolved STEM image in Figure 3F, which shows that a type I_1_ BSF (a single layer of cubic inclusion within the wurtzite crystal, i.e., a horizontal shear) can be present alongside the precipitate.

Also, in Figure 3F, the atomic structure of the precipitate clearly resembles a body‐centered tetragonal indium structure viewed down the [111] direction (right inset), and it differs from the wurtzite lattice (left inset). These crystal imperfections are visualized through the larger field‐of‐view geometric phase analysis (GPA) strain maps, where the dilation *D_xy_* map indicates the larger lattice parameter of the indium lattice, and the shear *ε_xy_* map demonstrates the horizontal translation of the basal planes in forming the stacking faults. Another example of such a defect bound by two precipitates is shown in Figure S9.

However, we note that the identity of these In‐precipitate‐linked defects could be more complex. Figure 3G now shows the atomic structure of a similar defect, viewed from the top down (larger field of view in Figure S9). Here, we observe the crystal on the left side of the image first translating horizontally, forming a prismatic stacking fault (likely bounding the BSF). However, a second diagonal translation (also along another <11$\bar{2}$0> direction) further occurs, which then resembles an inversion domain [36]. More examples are presented in Figure S10. Due to the different sample thickness requirements for CL and atomic imaging, we are unable to determine which of these different defects (stacking faults, inversion domains, or a combination of both) contribute to the spread of emissions between 500 and 565 nm. However, performing CL measurements on a separate LED sample, which contains only BSFs, shows a similar 80 nm blueshift from the target QW wavelength (Figure S11), indicating that BSFs could act as radiative recombination centers.

### 2.2 Nanoscale Compositional Inhomogeneities

We now demonstrate that APT can complementarily measure local compositional inhomogeneities within single QWs to explain their inhomogeneous emissions at 550 nm and above 600 nm. Figure 4A shows a measured 3D APT volume with 5 low and 3 high In‐content QWs; the left half is again occupied by a V‐pit. The relative indium compositional profile is plotted in Figure 4B, alongside the relative In‐signal acquired via EELS. Both results show that the high In% QWs contain roughly double the amount of indium as the low In% QWs. In our previous work [38], we have demonstrated the challenges in using cross‐sectional imaging and spectroscopy methods such as STEM‐based EELS for extracting compositional inhomogeneities. After all, typical TEM and STEM cross‐sectional samples are a few tens of nanometers thick and project the elemental signal throughout that sample thickness. For homogeneous samples, that would not be an issue; however, when the chemical elements are segregated in volumes much smaller than the sample thickness, these local variations are difficult to quantify with 2D projection techniques such as EELS or EDS. Figure 4C shows two examples of this effect, where EELS indium maps are presented from a high In% QW (top) and from a low In% QW (bottom). Both QWs appear to have a uniform indium distribution. However, this is a result of projected clusters that overlap in the projected sample thickness.

On the other hand, APT is not a projection technique; rather, it natively measures the distribution of chemical elements in three dimensions. For the same QWs as shown in the projected EELS maps, the 3D indium density maps measured by APT are presented in Figure 4D. For the high In% QW (top), the local fluctuations in the indium composition now become apparent. In contrast, the more homogeneous distribution of indium in the low In% QWs (bottom) can robustly be confirmed.

Further APT measurements confirm these observations; Figure 4E shows results from a sample that was cut perpendicular to the QWs. From this perpendicular, cross‐sectional APT sample and from the planar one, the indium density in *individual* QWs can be extracted, examples of which are presented in Figure 4F for low In% QWs, and in Figure 4G for high In% QWs. There is a striking difference between these sets, with the indium distribution in the high In% QWs being far from uniform. In the semi‐circular cross‐sectional maps, we observe indium‐rich precipitates, as in Figure 3, appearing as dark red clusters. In addition, in the square indium maps from the perpendicular sample, the compositional inhomogeneities of the high In% QWs are apparent. Analysis was also performed on the sidewall QWs, where the differences are less stark (Figure S12). It is thus likely that these compositional variations in the high In‐content *c*‐plane QWs contribute to the optical inhomogeneity measured with STEM CL. We will next suggest a possible origin with the help of DFT calculations.

### 2.3 Origins of Compositional Segregation

**FIGURE 5:**
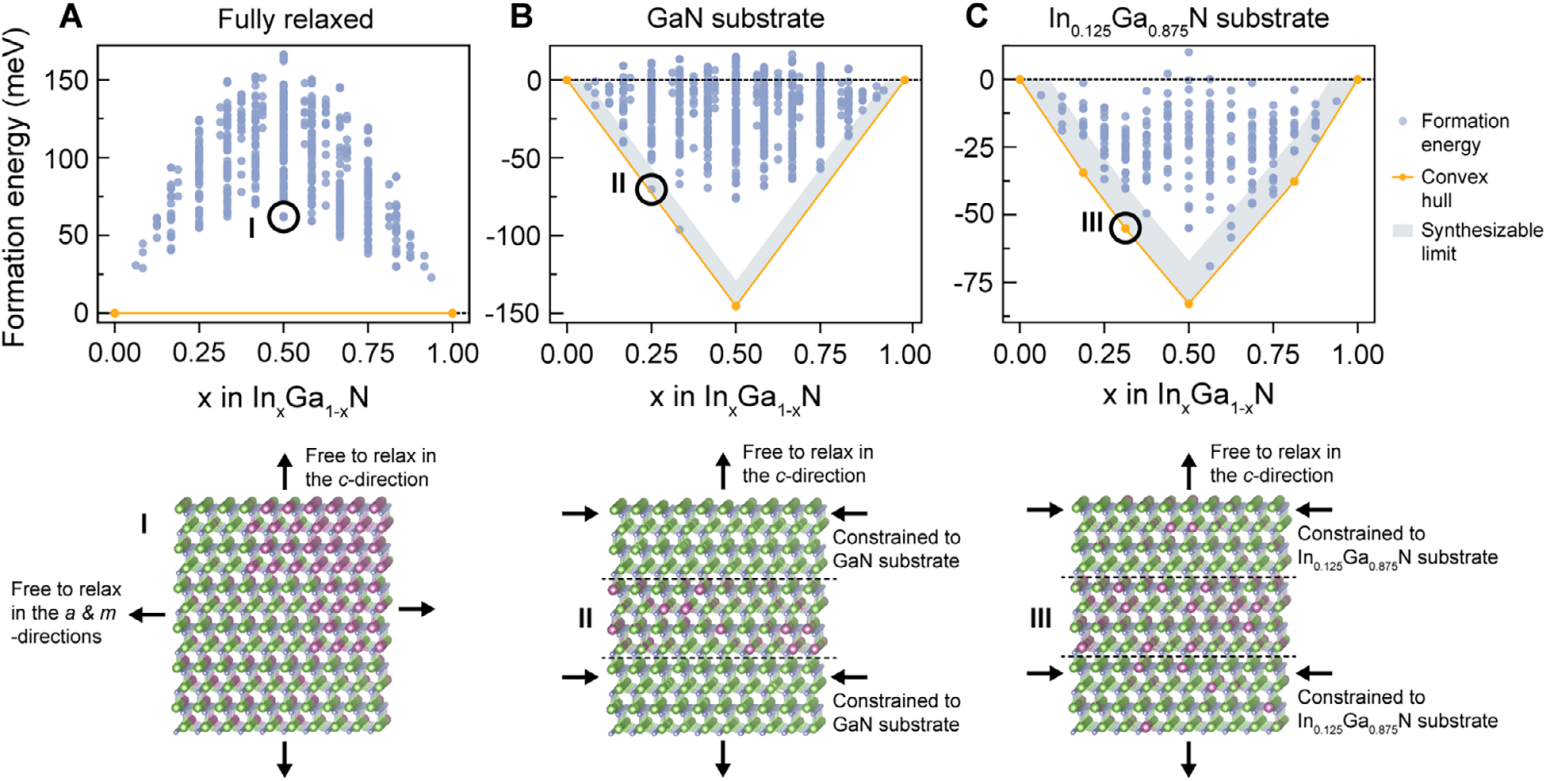
Computed formation energy of In_x_Ga_1−x_N films as a function of strain state and elemental composition. (A) Without lateral constraint from a substrate, (B) epitaxially grown on a GaN substrate, and (C) epitaxially grown on In_0.125_Ga_0.875_N. Each blue or orange dot represents a specific configuration of the alloy, where indium atoms substitute for gallium at different locations; the orange dots denote the ground‐state energies for each constrained model. The orange lines show the convex hull constructions, derived from SCAN‐based DFT calculations. The synthesizable limit range is marked by the gray shading, taken as 15 meV/f.u. above the convex hull. The atomic models in the bottom panel of respective convex hulls visually depict the different constraints used in the calculations. The specific models illustrating the constraints in the bottom panels are marked in the respective convex hulls in the top panel of each strain state.

As with most alloys, the two end members of In_x_Ga_1 − x_N, GaN and InN, are not fully miscible, and several intermediate compound phases exist. The stability of an alloy is often assessed by examining the miscibility of its end members using the convex hull formalism. The convex hull, which visually summarizes the results from a number of DFT calculations at different In_x_Ga_1 − x_N compositions, indicates whether a compound is stable or will decompose into a combination of varying compounds, as shown in Figure 5. Phases with the most negative formation energies, which—together with metastable phases very close to the convex hull (< 15 meV/formula unit)—are thermodynamically stable [55], while phases well above the convex hull are considered to be unstable. To ensure that all ground states are included in the convex hulls, the DFT‐calculated convex hulls were fitted to a cluster expansion, and a simulated annealing procedure at a fixed chemical potential was performed, as described in the Methods section and illustrated in Figure S3.

In the convex hull (totaling 576 DFT calculations) built with In_x_Ga_1 − x_N ordering models whose atomic positions and shapes are fully relaxed, and the entire volume of the supercell allowed to relax in all directions (Figure 5A), the In_x_Ga_1 − x_N alloy intrinsically shows a high propensity for phase separation at any concentration, as also described in our previous work [38]. The visual representation of the degree of freedom during the DFT calculations for reconstructing the fully relaxed convex hull is shown in the bottom panel of Figure 5A. However, this situation appears unrealistic. In contrast, when the basal plane of a configuration is constrained to a fixed lattice that simulates the effect of a substrate [56], it results in stable intermediate compositions, as seen from the convex hull construction in Figure 5B,C.

Although the first In_x_Ga_1 − x_N atomic layer grown epitaxially on GaN will closely follow the fixed GaN lattice, that may not be the case for subsequent In_x_Ga_1 − x_N layers. In the growth direction, there will be an increased relaxation of the lattice toward that of In_x_Ga_1 − x_N. To simulate this effect of changing substrate parameters, we performed DFT calculations with the basal plane of the supercell model constrained to that of both GaN (Figure 5B) and In_0.125_Ga_0.875_N (Figure 5C). These constraints and the axis of freedom are visually shown in the bottom panel of Figure 5B,C, using the respective structures marked in the convex hulls. The convex hulls for strain states of In_x_Ga_1 − x_N films grown on GaN substrate (Figure 5B) and on In_0.125_Ga_0.875_N substrate (Figure 5C) are constructed using 608 and 234 DFT calculations, respectively. These situations partially mimic realistic epitaxial growth conditions for high In‐content QWs, for which x‐ray diffraction (XRD) reciprocal space maps have indeed shown slight strain relaxation [53, 57, 58]. We emphasize that the actual strain states in our QWs do not reach this value; doing so would have required the use of underlying In_x_Ga_1 − x_N pseudo‐substrates [49, 54, 59]. However, it appears computationally impractical to perform equivalent calculations for lower In% In_x_Ga_1 − x_N substrates due to exponentially larger supercells required. Previous works have predicted a relationship between elastic strain and phase separation by modelling uniform compression of the epitaxial layer with respect to a fully strained GaN substrate [60]. Our present first‐principles approach builds on this perspective and predicts insightful trends for the case of partially relaxed substrates (Figure 5C and the case of an In_0.25_Ga_0.75_N substrate in Figure S13), the stability of In_x_Ga_1 − x_N phases from dynamic strain relaxation during growth, as summarized in the next paragraph.

When the underlying substrate is partially relaxed during growth, phases with compositions of In ≈ 22% and 33% become fully stabilized, as shown in Figure 5C. Furthermore, it is interesting to note that a larger number of high In‐content configurations (including one at 38%) now fall within the synthesizable limit. This trend can also be seen in the more extreme case of the In_0.25_Ga_0.75_N substrate constraint in Figure S13, where the formation energies of phases near these compositions (between 25% and 38% In) are further lowered toward the convex hull. These results provide a possible explanation for the inhomogeneous emission observed in device architectures using relaxed In_x_Ga_1 − x_N pseudo‐substrates [49, 54, 59]. In addition, in device structures with strain engineering measures in place—in our case, AlN strain‐compensating capping layers—unintentional local In_x_Ga_1 − x_N phases may be stabilized, leading to the compositional segregation that we observe with APT, and correspondingly explaining the STEM CL optical inhomogeneities.

## 3 Conclusion

This work demonstrates that phase segregation in In_x_Ga_1 − x_N QWs can lead to broadened emission due to the formation of nanoscale optical inhomogeneities. In practice, inhomogeneous emission is not always observed for similar QW deposition conditions on differently strained substrates [49, 53]. Our results suggest that both the underlying strain and the composition of the In_x_Ga_1 − x_N layer influence whether phase segregation occurs. The prevention of phase separation in GaN‐based HEMT and LEDs appears challenging, but can be addressed by aiming for the thermodynamically stable ground‐state compositions for the expected strain conditions. A well‐designed substrate and strain engineering layers, together with a carefully chosen indium percentage, can then lead to a stable In_x_Ga_1 − x_N phase for homogeneous, long‐wavelength emission.

In summary, by using scanning transmission electron microscopy‐based cathodoluminescence, we observed nanoscale optical inhomogeneity within individual In_x_Ga_1 − x_N quantum wells, which are targeted for amber‐to‐red emission. These emissions—550, 580, and 620 nm—correspond to some degree to the injection current blueshifted emissions in recent red In_x_Ga_1 − x_N LED reports. Our atom probe tomography measurements directly show that these optical inhomogeneities result from the compositional segregation of high‐indium‐content quantum wells, which does not occur in low‐indium‐content quantum wells. Density functional theory calculations further suggest that in‐plane strain can have a stabilizing effect on phases that roughly correspond to the observed emissions. On the basis of our results, we propose that not just strain‐engineering, but also the proper selection of the In‐composition (where growth parameters will then need to be taken into account) will be key for efficient homogeneous, long‐wavelength In_x_Ga_1 − x_N LEDs and next‐generation HEMTs.

## 4 Experimental Section

### 4.1 III‐Nitride Device Architecture

The III‐nitride layers were epitaxially deposited through metal–organic chemical vapor deposition (MOCVD) on 8″ Si (111) substrates (thickness∼1 mm) in an AIXTRON CRIUS close‐coupled‐showerhead (CCS) reactor with trimethylaluminium (TMAl), trimethylgallium (TMGa), trimethylindium (TMIn), and NH_3_ as precursors, and H_2_/N_2_ as carrier gases. The compositions of the light‐emitting InGaN QWs were varied by tuning the chamber temperature—745°C, 715°C, and 685°C for the architecture shown in Figure 1, and 760°C and 630°C for the architecture shown in Figure 2. Below the QWs, low In‐content (∼ In_0.07_Ga_0.93_N) V‐pit initiation layers were grown to intentionally open V‐pits from threading dislocations. Above the QWs, an AlGaN electron blocking layer (EBL) prevents carrier overflow to the p‐GaN layer, capping the LED structure.

### 4.2 TEM Sample Preparation

Electron‐transparent foils were prepared using the conventional mechanical polishing approach (Allied MultiPrep System) combined with broad Ar^+^ ion milling. Prior to polishing, the LED samples are first processed into ‘sandwiches’, where two pieces from the wafer (3 × 1 mm) are glued (EpoxyBond 110) face‐to‐face from their film ends. The sandwiches were polished sequentially using diamond lapping films of grades 35, 15, 9, 3, 1, and 0.1 µm film. For Ar^+^ ion thinning to electron transparency, a Fischione 1051 TEM mill was used, with milling performed at −120°C. To minimize the formation of amorphous layers, the final acceleration voltage and the angle of incidence were kept at 0.8 kV and 5° (grid‐side)/4° (sample side), respectively.

### 4.3 STEM CL and EELS Acquisition and

STEM CL data were acquired with both a FEI Titan G1 equipped with a Gatan Vulcan CL liquid nitrogen cooled holder with ellipsoidal collection mirrors, as well as a Thermofisher Spectra 300 equipped with an Attolight Mönch with a parabolic mirror. A specially designed Mel‐Build liquid N_2_ cooled holder was used in conjunction with the Attolight Mönch setup. STEM EELS data were acquired with a JEOL JEM‐ARM200CFEG equipped with a fifth‐order ASCOR aberration‐corrector, and a Gatan GIF Quantum EELS detector, operated at 80 kV. For core‐loss elemental edge analysis, an EELS collection semi‐angle of ∼77 mrad, and energy dispersion of 0.40 eV/channel were used. *Z*‐contrast HAADF and strain‐contrast LAADF images were captured with annular detectors with collection semi‐angles: *β* = 68−280 and 30−120 mrad, respectively. An *α* = 31 mrad convergence angle was used for the acquisition of images.

### 4.4 APT Parameters

Laser‐assisted APT was carried out using a CAMECA Invizo 6000. Samples were prepared in both the top‐down (planar) and cross‐sectiona orientation through FIB lift‐out with a final 1 kV cleaning step. For the analysis, all samples were cooled down to a temperature of 30 K. The experimental data were collected at a laser wavelength of 257.5 nm, pulse rate of 200 kHz, laser power between 25 fJ and 0.5 pJ, and a detection rate of 2%. APT data were reconstructed using the Integrated Visualization & Analysis Software (IVAS) by cross‐referring to the TEM cross‐section images for layer thicknesses and spacing. By distinguishing the V‐pits, analysis was conducted by extracting individual *c*‐plane QWs of the LED under both planar and cross‐section orientations.

For the calculations of the relative indium fraction of the QWs in the *c*‐direction, the indium count was summed across an area of 9350 nm^2^ at the interior of the *c*‐plane QW region in the cross‐section sample and divided by the total number of metal‐site ions at each *z*‐step of 0.1 nm. The projected In density maps displayed as cuboids in Figure 4D, F,G were likewise extracted from roughly the interior of the *c*‐plane QW region in the samples.

### 4.5 DFT Calculations

Density functional theory (DFT) employing the sufficiently constrained and appropriately normed (SCAN) meta‐GGA exchange‐correlation functional [61] was performed combining plane waves and projector augmented‐wave (PAW) potentials to describe the InGaN wave functions, as implemented in VASP [62, 63] In PAWs, the following electrons were treated explicitly: In 4d^10^5s^2^5p^1^ (06Sep2000), Ga 3d^10^4s^2^4p^1^ (06Jul2010), and N 2s^2^2p^3^ (08Apr2002). A plane‐wave basis with an energy cut‐off of 520 eV and an 8 × 8 × 5 Gamma‐centered Monkhorst‐Pack *k*‐point mesh was used for the primitive cell of GaN (with two formula units and *P6_3_mc* space group). For the larger supercells, the *k*‐point meshes were adjusted to achieve the same sampling of the first Brillouin zone. The total energy was converged within 10^−5^ eV/cell, and the interatomic forces (and stresses) were converged to less than 10^−2 ^eV/Å (0.29 GPa).

The convex hulls were fitted using a cluster expansion model, followed by a simulated annealing procedure accessed by grand‐canonical Monte Carlo simulation at a fixed chemical potential to find any missing ground states. If any such ground state structure is found, the DFT energy for this structure is calculated and incorporated again into the cluster expansion model. This procedure was applied iteratively until no further ground state structures were found.

## Author Contributions

J.‐Y.C., T.P.M., S.G., P.C., and M.B. conceptualized and designed the experiments. J.‐Y.C. and G.J.S. prepared the TEM samples and performed the STEM and EELS measurements under the guidance of S.J.P. Z.M., T.E.G., and J.W. performed the STEM CL measurements. K.P.R. and I.M. prepared the APT samples and performed the APT experiments. L.Z. fabricated the LEDs. T.P.M. performed the DFT calculations under the guidance of P.C. J.‐Y.C., T.P.M., and M.B. wrote the manuscript with input from all authors.

## Funding

This research is supported by the Ministry of Education, Singapore, under its AcRF Tier 2 programme (MOE‐T2EP50122‐0016). Work performed at the Center for Nanoscale Materials, a U.S. Department of Energy Office of Science User Facility, was supported by the U.S. DOE, Office of Basic Energy Sciences, under Contract No. DE‐AC02‐06CH11357. P.C. acknowledges the Welch Foundations under grants L‐E‐001‐19921203, and E‐2227‐20250403.

## Conflicts of Interest

K.P.R. and I.M. are employed by CAMECA Instruments, the manufacturer of the APT instruments that were used for some of experiments in this work. The other authors declare that they have no competing interests.

## Supporting information




**Supporting File 1**: advs76414‐sup‐0001‐SuppMat.docx.

**Supporting File 2**: advs76414‐sup‐0002‐VideoS1.mp4.

## Data Availability

All data needed to evaluate the conclusions in the paper are present in the paper and/or the Supplementary Materials.
