# Supplementary material for: Nanoscale Optical Inhomogeneities From Compositional Segregation Within Individual GaN‐on‐Si Quantum Wells
Source: Adv Sci (Weinh). 2026 Jul 6:e76414. Online ahead of print. doi: 10.1002/advs.76414 (PMC13335705; doi:10.1002/advs.76414)
Supplement: Supplementary file 1 — Supporting File 1: advs76414‐sup‐0001‐SuppMat.docx. [file ADVS-9999-e76414-s001.docx]

Supporting Information

**Nanoscale Optical Inhomogeneities from Compositional Segregation within Individual GaN-on-Si Quantum Wells**

*Jing-Yang Chung,^1,2,^*^†^ *Tara P. Mishra,^1,2,^*^†^ *Zackaria Mahfoud,^3,^*^†^ *Thomas E. Gage,^4^* *Jianguo Wen,^4^* *Katherine Rice,^5^* *Li Zhang,^2^ Govindo J. Syaranamual,^2^ Stephen J. Pennycook,^1,2^*
*Silvija Gradečak,^1,2^ Pieremanuele Canepa,^1,2,6,7^ and Michel Bosman^1,3,*^*

^1^Department of Materials Science and Engineering, National University of Singapore, 117575 Singapore

^2^Singapore-MIT Alliance for Research and Technology, 138602 Singapore

^3^Institute of Materials Research and Engineering, Agency for Science, Technology and Research (A*STAR), 138634 Singapore

^4^Center for Nanoscale Materials, Argonne National Laboratory, Lemont, Illinois 60439, United States

^5^CAMECA Instruments, Inc., Madison, Wisconsin 53711, United States

^6^Department of Electrical and Computer Engineering, University of Houston, Houston, Texas 77204, United States

^7^Texas Center for Superconductivity, University of Houston, Houston, Texas 77204, United States

^†^ These authors contributed equally: Jing-Yang Chung, Tara P. Mishra, Zackaria Mahfoud

* Corresponding Author: msemb@nus.edu.sg

1. **Experimental Details**
   1. **III-nitride device architecture**

LED devices in this study were prepared in the same manner reported previously by Zhang *et al.*^1^ The III-nitrides layers were epitaxially deposited through metal organic chemical vapor deposition (MOCVD) on 8” Si (111) substrates (thickness~1mm) in an AIXTRON CRIUS^®^ close-coupled-showerhead (CCS) reactor with trimethylaluminium (TMAl), trimethylgallium (TMGa), trimethylindium (TMIn) and NH_3_ as precursors, and H_2_/N_2_ as carrier gases for GaN and InGaN respectively. The compositions of the light-emitting InGaN quantum wells (QWs) were varied by tuning the chamber temperature — 745°C, 715°C, and 685°C for the architecture shown in **Figure 1** in the **Main Text**, and 760°C and 630°C for the architecture shown in **Figures 2** in the **Main Text**. Below the QWs, low In-content (~ In_0.07_Ga_0.93_N) V-pit initiation layers were grown to intentionally open V-pits from threading dislocations. Above the QWs, an AlGaN electron blocking layer (EBL) prevents carrier overflow to the p-GaN layer capping the light-emitting diode (LED) structure.


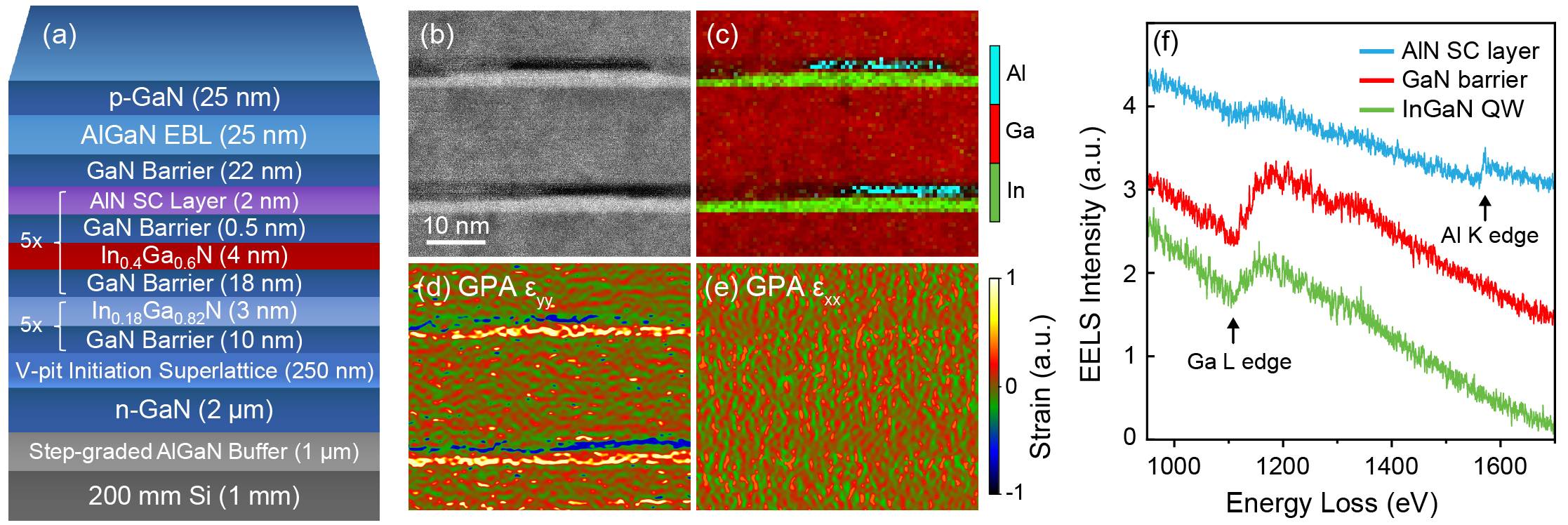


**Figure S1.** (a) Schematic illustration of the GaN-on-Si structure with AlN capping layers above the high In-content QWs. (b) HAADF image of two InGaN/GaN/AlN stack, and the corresponding (c) EELS composite map showing the Al, In and Ga elemental distributions. (d)–(e) GPA strain maps processed from (b), illustrating the out-of-plane strain ε_yy_ and in-plane strain ε_xx_ of the layers, respectively. (f) Core loss spectra comparing the AlN capping layer (in blue), GaN barrier (in red), and InGaN QW (in green).

**1.2 TEM sample preparation**

Electron transparent foils were prepared using the conventional mechanical polishing approach (Allied MultiPrep™ System) combined with Ar+ ion milling in a Fischione TEM mill.^2^ Prior to polishing, the LED samples are first processed into ‘sandwiches’, where two pieces from the wafer (3x1 mm) are glued (EpoxyBond 110™) face-to-face from their film ends. Rough polishing was made with a 35 μm grade diamond lapping film, and finer polishing with 15 μm, 9 μm, 3 μm, and 1 μm films, which brings the sample thickness down to 90 μm, 50 μm, 30 μm, ~20-10 μm, respectively. Upon the sample reaching reddish-brown transparency, final polishing was conducted using a 0.1 μm film. For Ar+ ion thinning to electron transparency, a Fischione 1051 TEM mill was used, with milling performed at -120°C. To avoid the formation of amorphous layers, the final acceleration voltage and the angle of incidence were kept at 0.8 kV and 5° (grid-side) / 4° (sample side), respectively.

**1.3 STEM CL and EELS acquisition and data processing**

STEM CL data were acquired with both a FEI Titan G1 equipped with a Gatan Vulcan CL liquid nitrogen cooled holder with ellipsoidal collection mirrors, as well as a Thermofisher Spectra 300 equipped with a Attolight Mönch with a parabolic mirror. A specially designed Mel-Build liquid N_2_ cooled holder was used in conjunction with the Attolight Mönch setup. Ejected photons are focused into optical fibres and collected with a back-illuminated CCD, recording the CL spectrum per pixel. Prior to data analysis, the CL spectra were processed to remove systematic experimental noise by averaging the signal per pixel in a map taken at a vacuum region, then subtracting the averaged spectrum across the desired CL dataset. For the dataset shown in **Figure 2** in the **Main Text**, this method was likewise used to remove the well-reported broad yellow donor-acceptor pair (DAP) defect emission resulting from Ga vacancies coupled to oxygen complexes,^3^ which signature remains mostly constant throughout the entire spectrum map (see **Figure S2a**). For this case, the background was instead taken at a pure GaN region (red curve in **Figure S2a**). Fitting was first conducted in terms of photon energy (**Figure S2b**), then converted to a linear wavelength axis (**Figure S2c**).


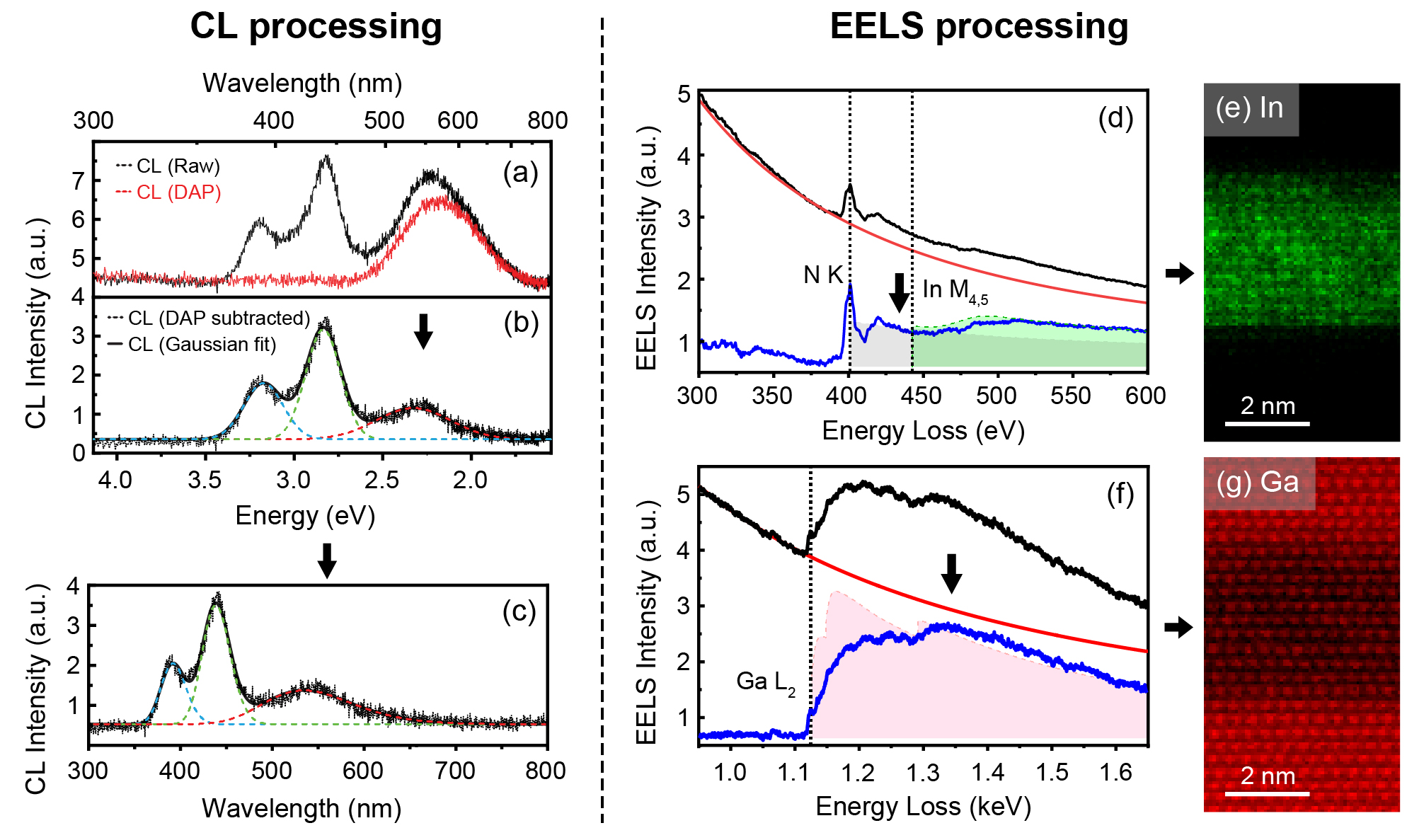


**Figure S2.** Example STEM CL data acquired at a V-pit region. (a) Raw spectrum (in black) represented in terms of photon energy. The red curve represents the DAP emission constant throughout the hyperspectral map. (b) The DAP-subtracted spectrum fitted with gaussian curves. (c) The fitted spectrum converted back to a linear wavelength scale. (d) STEM BF image of the V-pit, and (e)–(g) corresponding CL wavelength-filtered spectral maps at 380 nm, 440 nm, and 550 nm, respectively.

STEM EELS data were acquired with a JEOL JEM-ARM200F equipped with a cold field emission gun (CFEG) and a fifth-order ASCOR aberration-corrector, operated at 80 kV. Z-contrast high-angle annular dark-field (HAADF) images were captured with an annular detector with collection semi-angles: *β* = 68−280 mrad, while strain-contrast low-angle annular dark-field (LAADF) images were captured with an annular detector with collection semi-angles: *β* = 30−120 mrad. An *α* = 31 mrad convergence angle was used for the acquisition of images. For EELS acquisition, dual Gatan GIF quantum energy filters with collection angle ∼77 mrad, and energy dispersion set at 0.40 eV/channel for core-loss elemental edge analysis was used. In elemental maps were extracted using the In M_4,5_ edge (see **Figures S2d-e**). Although not shown in this paper, the corresponding Ga elemental maps were simultaneously acquired and extracted using the Ga L_2_ edge for cross-comparison with the In maps (see **Figures S2f-g**).

**1.5 APT parameters**

APT was carried out using a CAMECA Invizo® 6000. Samples were prepared in both the top-down (planar) and cross-section orientation through FIB lift-out with a final 1 kV cleaning step. For the analysis, all samples were cooled down to a temperature of 30 K. The experimental data are collected at a laser wavelength of 257.5 nm, pulse rate of 200 kHz, and laser power of 0.5 nJ. APT data were reconstructed using the Integrated Visualization & Analysis Software (IVAS) 3.6.8. By distinguishing the V-pits, analysis was conducted by extracting individual *c*-plane QWs of the LED under both planar and cross-section orientations.

For the calculations of relative indium-fraction of the QWs in the *c*-direction, the indium count was summed across an area of 9,350 nm^2^ at the interior of the *c*-plane QW region in the cross-section sample and divided by the total number of metal-site ions at each *z*-step of 0.1 nm. The projected In density maps displayed as cuboids in **Figures 4d** **and 4f–g** in the **Main Text** were likewise extracted from roughly the interior of the *c*-plane QW region in the samples.

**1.6 DFT calculations**

Density functional theory (DFT) employing the sufficiently constrained and appropriately normed (SCAN) meta-GGA exchange correlation functional^4^ was performed using a combination of plane-waves and projector augmented-wave (PAW) potentials to describe the InGaN wave-functions, as implemented in VASP.^5,6^ In the PAWs the following electrons were treated explicitly: In 4d^10^5s^2^5p^1^ (06Sep2000), Ga 3d^10^4s^2^4p^1^ (06Jul2010) and N 2s^2^2p^3^ (08Apr2002). A plane-wave basis with an energy cut-off of 520 eV and a 8x8x5 Gamma centered Monkhorst-Pack *k*-point mesh were used for the primitive cell of GaN (with 2 formula units and *P6_3_mc* space group). As for the larger supercells, the *k*-point meshes were adjusted to obtain the same sampling of the 1^st^ Brillouin zone. The total energy was converged within 10^–5^ eV/cell and the interatomic forces (and stresses) were converged to less than 10^­–2^ eV/Å (0.29 GPa).

The convex hulls were fitted to a cluster expansion model (**Figure S3**), followed by a simulated annealing procedure at a fixed chemical potential^7^ to find any missing ground states. The details of the cluster expansion fitting and simulated annealing are explained in detail in our previous work.^7^ If any such ground state structure is found, the DFT energy for this structure is calculated and incorporated again to the cluster expansion model. This procedure applied iteratively until no further ground state structures were found.


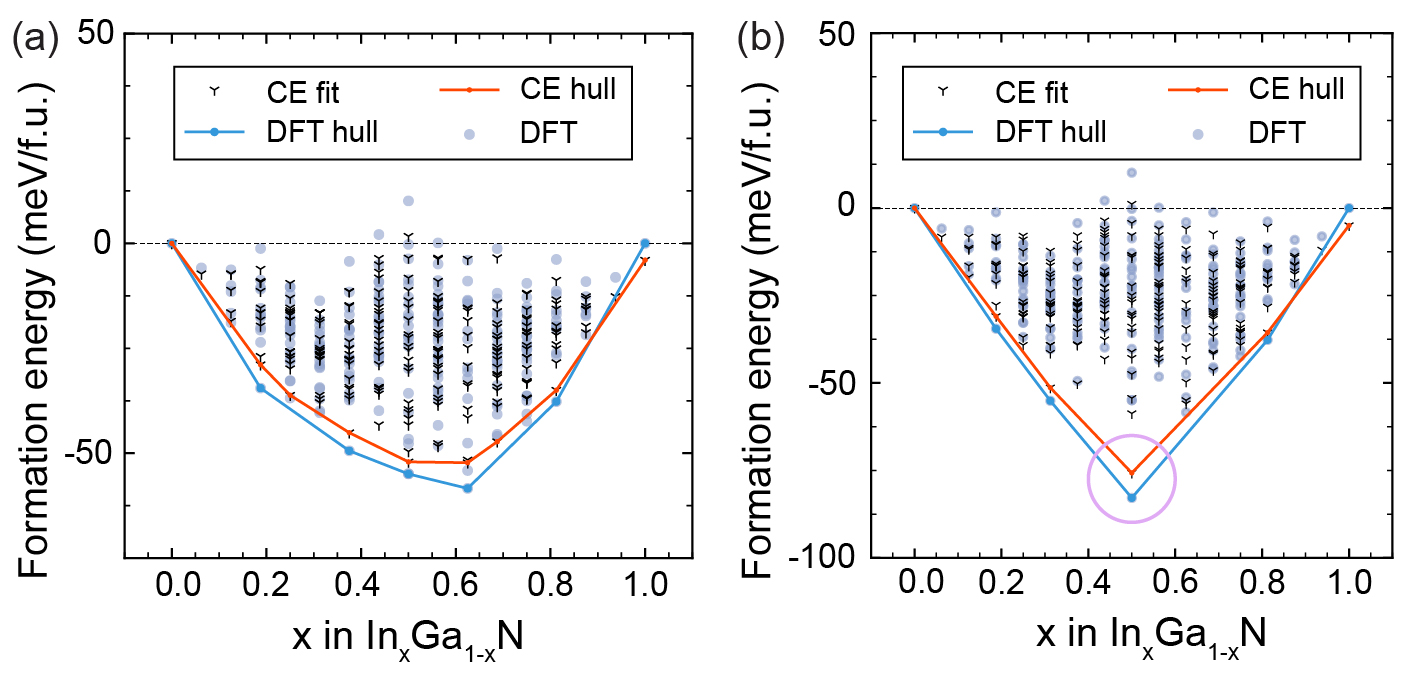


**Figure S3.** Illustration of the ground state finding algorithm for GaN substrate. (a) The initial cluster expansion used for simulated annealing at a fixed chemical potential. This process identified a new ground state structure at In=0.5. (b) The resulting ground state, highlighted by a pink circle, is shown alongside the final cluster expansion, which did not yield any additional ground states.

1. **Supplementary Figures**

**
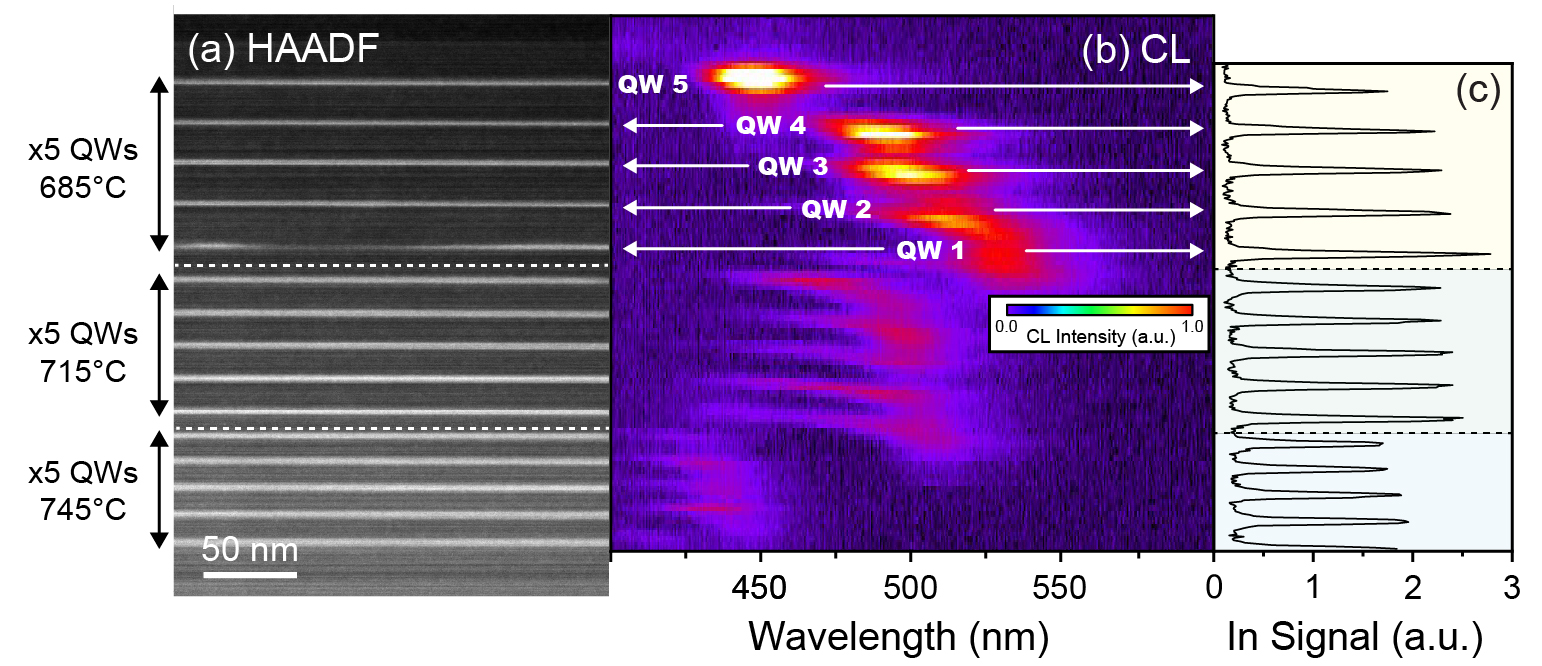
**

**Figure S4.** Cross-section STEM HAADF and 2D CL images of the structure in **Figure 1** in the **Main Text**. On the right of the figure, the integrated signal from the In Lα emission obtained from elemental dispersive X-ray spectroscopy (EDS) is shown for the various QWs. The blue-shifting of the upper five QWs deposited at 685°C could be attributed to the decrease in In% observed from EDS.


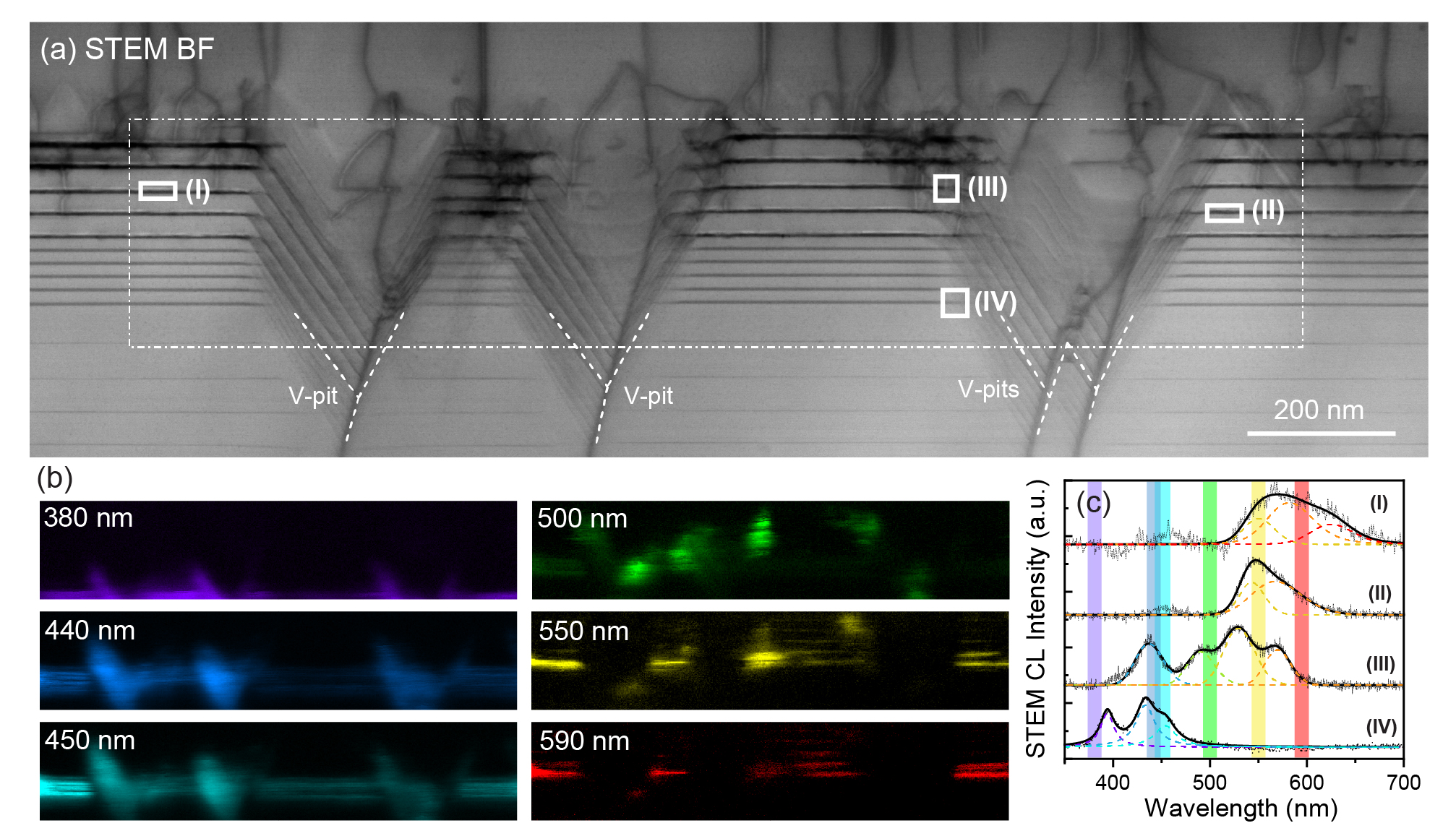


**Figure S5.** (a) Bright-field STEM image of the region where the hyperspectral map shown in **Figure 2** in the **Main Text** was taken. (b) shows the separated individual maps making up the polychromatic image in **Figure 2c** in the **Main Text**. (c) Extracted spectra from the region in (a). The color bands demonstrate the binned wavelength widths used to generate the monochromatic images in (b). The emission from the upper QW (shown in **I**) can be seen to consist of peaks roughly at 550 nm, 580 nm, and 625 nm.

**
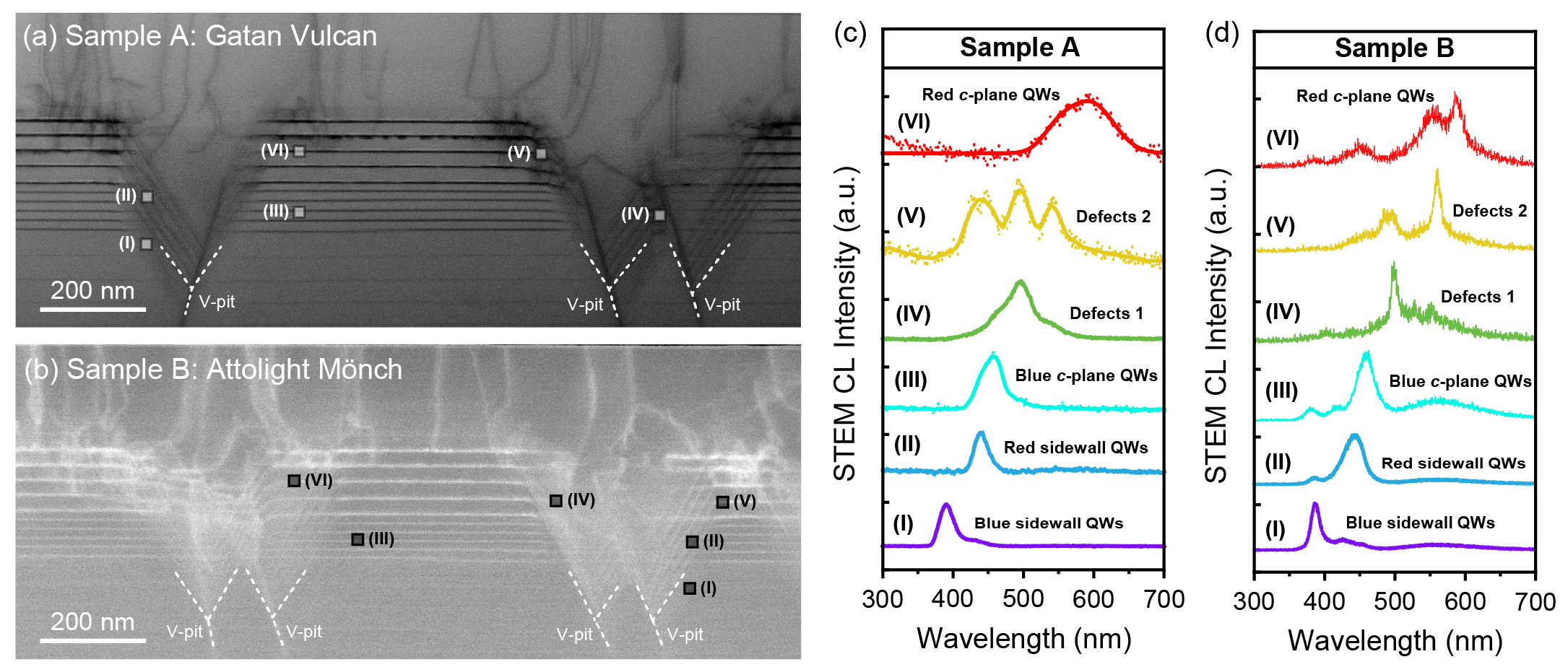
**

**Figure S6.** Further examples of CL measurements on similar regions to that shown in **Figure 2c** in the **Main Text**. (a) STEM BF image of a separate TEM sample in the [1$\bar{\text{1}}$00] zone-axis, and (c) corresponding point CL spectra acquired with the Gatan Vulcan CL system. (b) STEM LAADF image of another sample in the [11$\bar{\text{2}}$0] zone-axis, and (d) corresponding CL spectra acquired with the Attolight Mönch system. The spectra obtained are consistent with the results shown in **Figure 2f** in the **Main Text**.


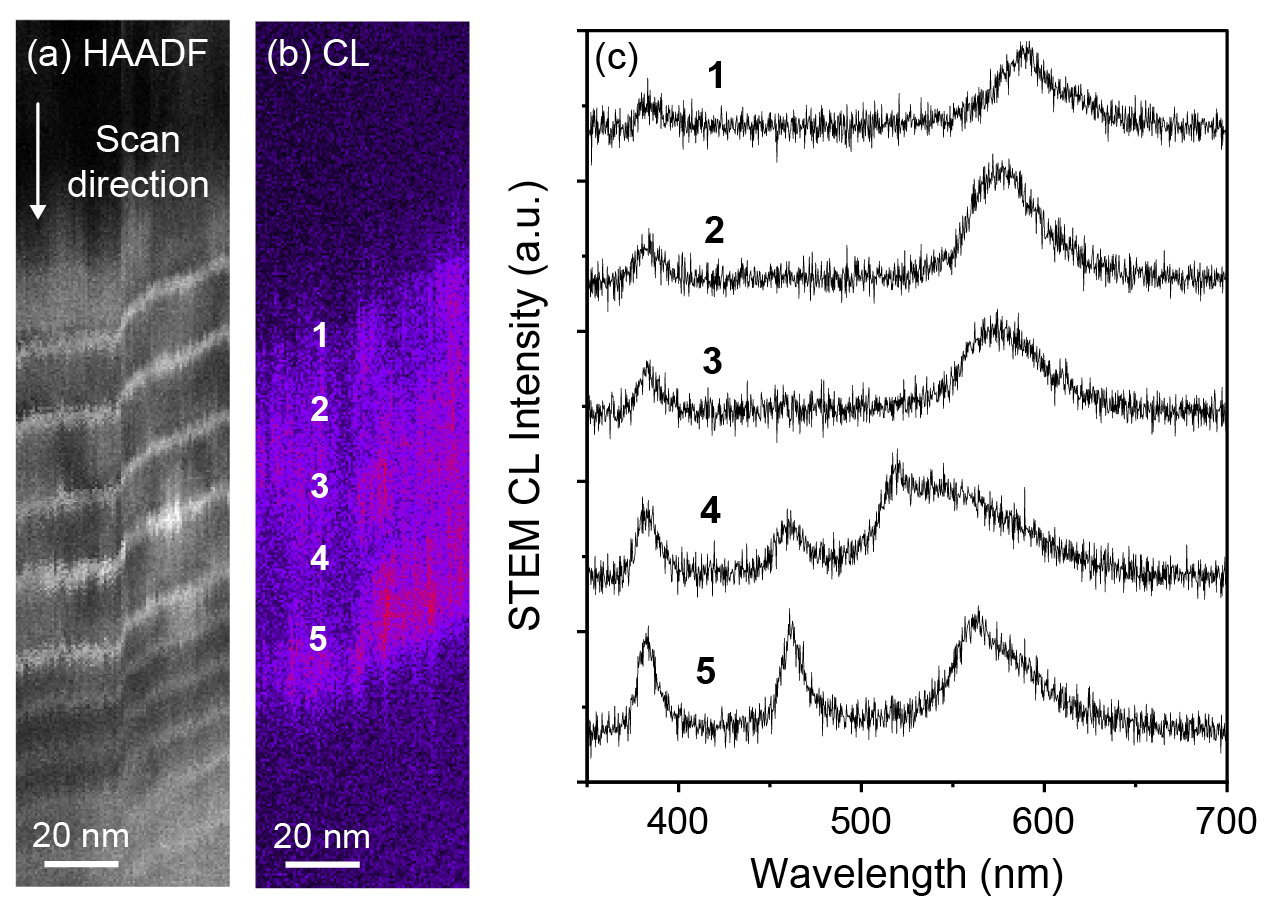


**Figure S7.** This figure helps to demonstrate that the observed emissions shown in the **Main Text** are unlikely to be due to beam-induced artifacts. In this dataset, the scanning direction of the electron beam is perpendicular to the QWs rather than parallel as that in the **Main Text**. (a) HAADF image of the high In-content QWs, and (b) corresponding CL map extracted at 580 nm show that all QWs emit at this wavelength. However, the extracted point spectra in (c) demonstrates—similar to that shown in **Figure 2f** in the **Main Text**—that broader emissions are detected for the lower QWs compared to the upper QWs, regardless of the scan direction. Notably in this dataset, sample drift occurred midway during the acquisition (as seen from the shearing of the QW), and the CL map likewise follows this drift direction.

**
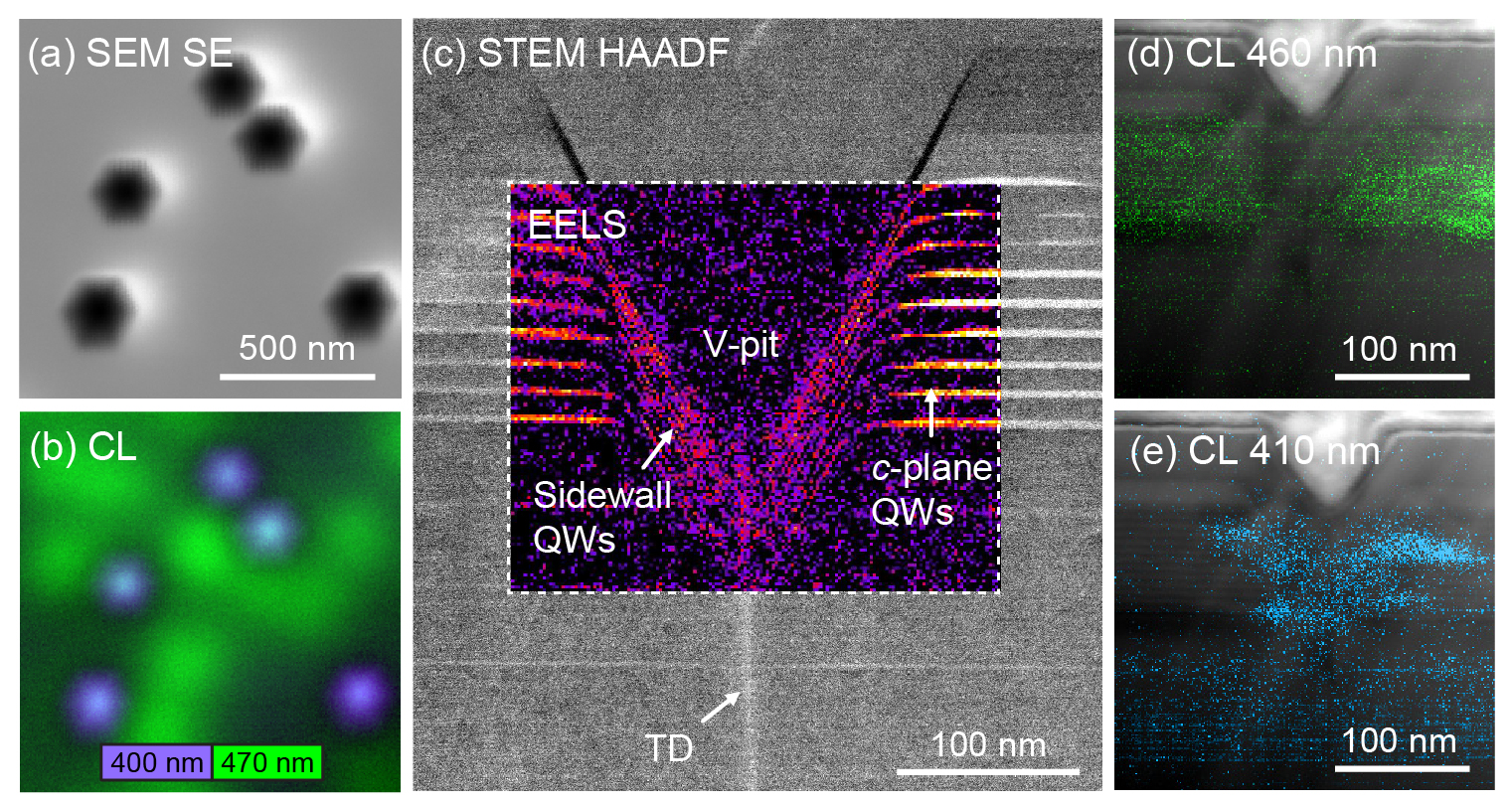
**

**Figure S8.** (a) SEM secondary electron (SE), and (b) composite monochromatic (400 nm in blue, 470 nm in green) CL on V-pits showing the emission wavelength distribution. Two-dimensional projection of a V-pit through: (c) STEM HAADF (the inset shows the elemental In EELS map where a decrease in the In-content at the sidewalls compared to the *c*-plane could be seen), and (d)–(e) STEM CL wavelength-filtered maps overlaid onto a BF image acquired at 460 nm and 410 nm, respectively.

**
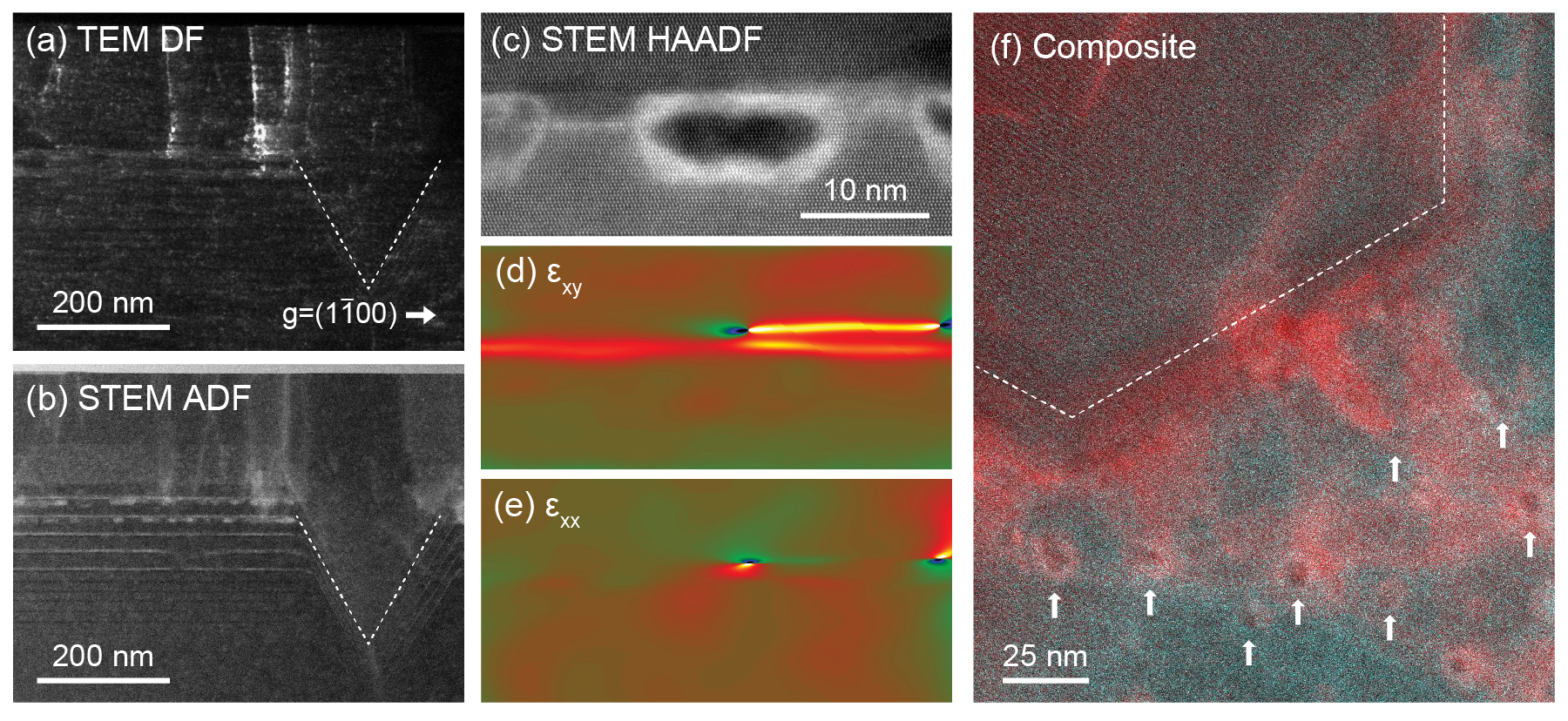
**

**Figure S9.** (a) TEM dark-field image, and (b) corresponding STEM ADF image showing the positions of the In-rich precipitates as bright spots along the upper two QWs. (c) HAADF image and corresponding (d) ε_xy_ shear and (e) ε_xx_ in-plane strain maps of a precipitate. The GPA maps show the defect to be a stacking fault (shear in the ε_xy_ map, similar to that in **Figure 3F** in the **Main Text**) bounded by two Frank partials (positive and negative strain in the ε_xx_ map). The strain from the precipitate does not show up in these strain maps as we selected the focus position to be slightly out of the precipitate region. (f) Composite STEM image comprising of HAADF (in cyan) and LAADF (in red) showing the positions of the precipitates (white arrows) linked by strain contrast lines (in red).

**
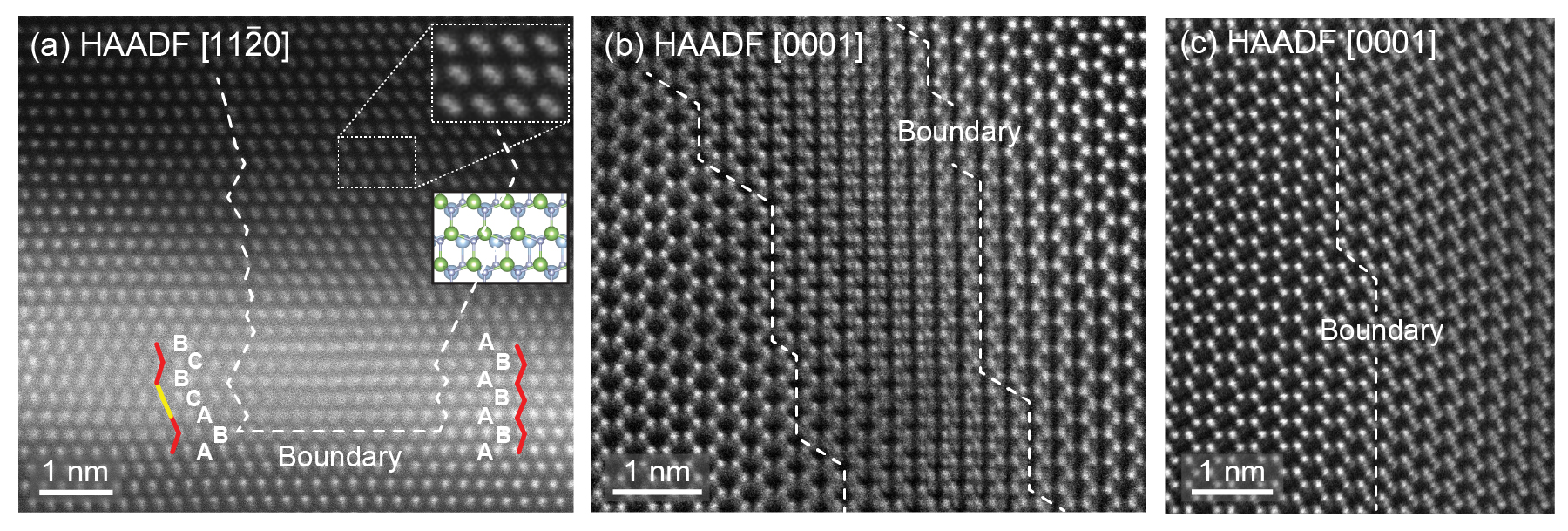
**

**Figure S10.** Further HAADF atomic images of the V-pit and precipitate-localized defects in the (a) cross-section [11$\bar{\text{2}}$0], and (b)–(c) plan-view [0001] directions. For (a) and (b), we presented these as examples of V-pit localized defects in the Supplementary of our previous study,^8^ which we also present here to link to the 500–565 nm optical emission observed. As we previously described, the left of the boundary in (a) contains two layers of cubic transition, changing the stacking sequence from ABABAB to ABACBC. Within the boundary, the overlap of atom columns reveals a vertical atom displacement, suggesting the defect identity could be a IDB bounded at its base to a BSF. Likewise, while (b) mostly takes the form of a prismatic stacking fault, (c) appears as an inversion domain instead.

**
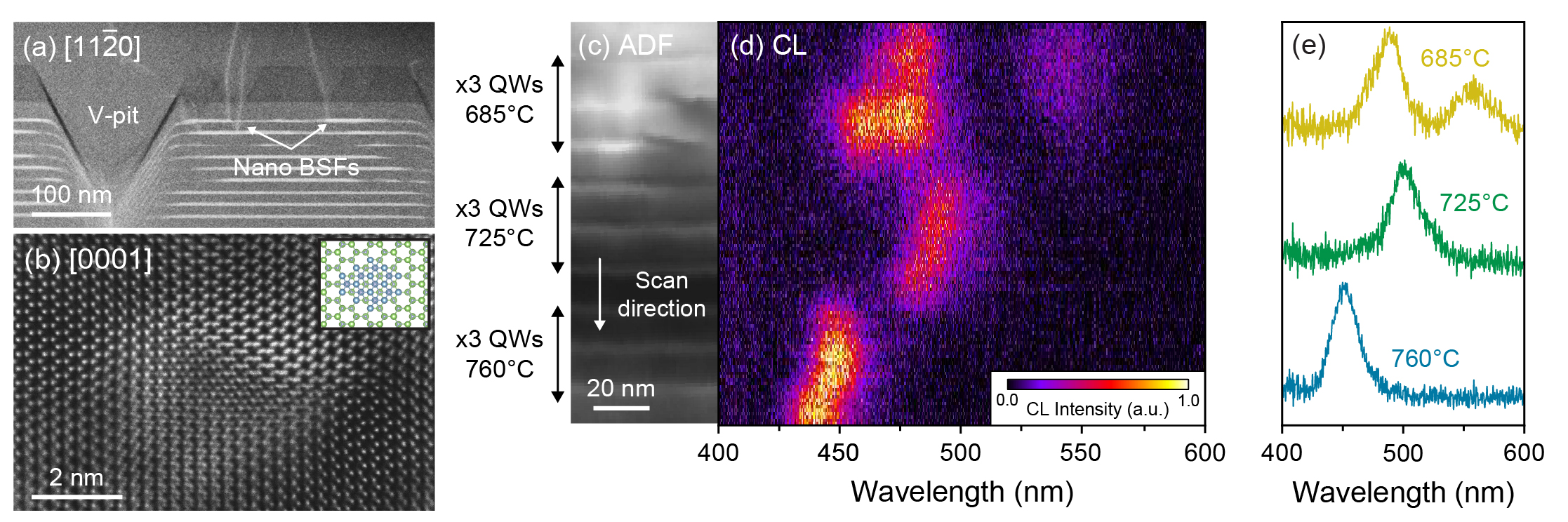
**

**Figure S11.** STEM CL performed on a sample with only BSFs also shows a blueshift from the target QW wavelength. (a) HAADF image of the QW structure with three different sets QWs, grown with decreasing temperatures of 760°C, 725°C, and 685°C. Threading defects appear from the upper set of QWs, which are revealed to be nano-sized BSFs from the top-down view in (b).This was also described in the Supplementary of our previous study.^8^ Likely due to these BSFs, the STEM CL map acquired from the region in (c) show a secondary 80 nm blueshifted peak from the target wavelength of 550 nm in the projected CL profile in (d) and extracted spectra in (e).

**
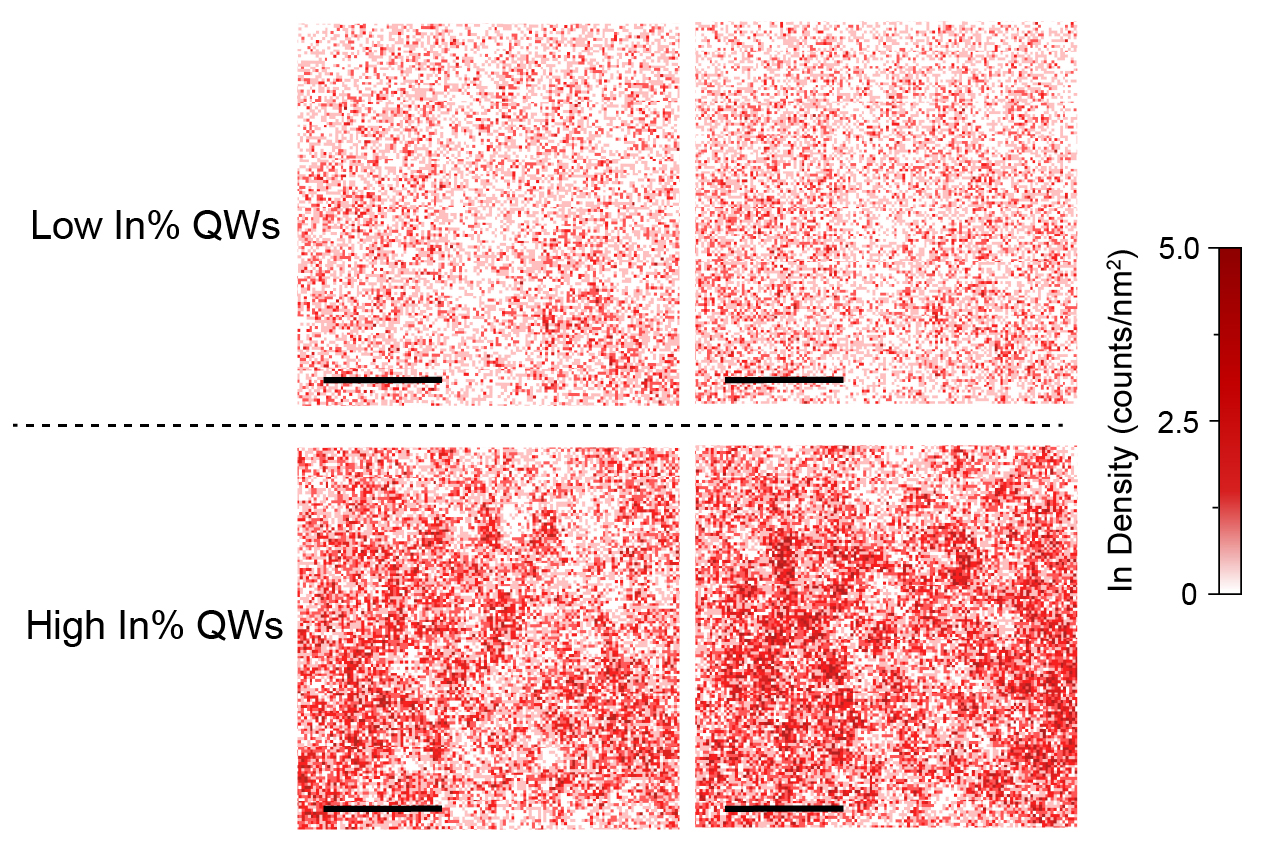
**

**Figure S12.** Projected APT indium density maps extracted from the low and high In-content sidewall {1$\bar{\text{1}}$01} semipolar QWs, similar to the analysis shown in **Figure 4** in the **Main Text**. The data was reconstructed from the V-pit region of the planar APT sample shown in **Figure 4E**. Due to the thinner widths of the sidewall QWs compared to the *c*-plane QWs, the maps were extracted from slices 4 nm thick, while the lateral binning was kept the same. The high In sidewall QWs display a higher degree of non-uniformity in the In distribution compared to the low In sidewall QWs. However, the inhomogeniety observed is noticeably less prevalent than that of the *c*-plane (**Figures 4F and G**) QWs, likely due to the overall lower In-concentration in the sidewall QWs. We note that previous reports have performed APT analysis on non-polar *a*-plane InGaN QWs, and have observed inhomogeneous distribution.^9,10^ Hence, this non-uniformity could arise from both the uneven strain distribution of the undulating AlN layer or the semipolar nature of the sidewall QWs. Scale bars are 20 nm.

**
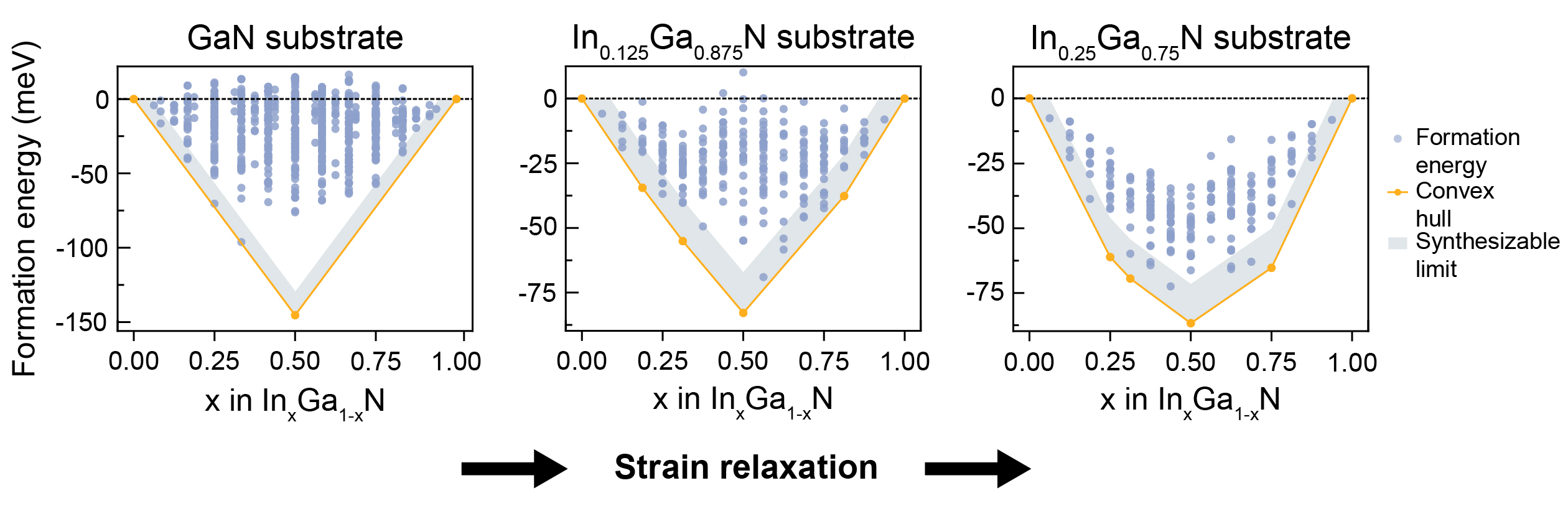
**

**Figure S13.** This figure demonstrates the trend in the formation energies of In_x_Ga_1−x_N when relaxing the strain further towards In_0.25_Ga_0.75_N lattice. Each blue or orange dot represents a particular configuration of the alloy with indium atoms substituting gallium at different locations; the orange dots denote the ground state energies for each constrained model. The orange lines show the convex hull constructions, derived from SCAN-based DFT calculations. The synthesizable limit range is marked by the grey background, taken as 15 meV/f.u. above the convex hull. An increasing number of phases fall within the synthesizable limit when the strain is further relaxed.

**References**

1. Zhang, L.; Lee, K. H.; Riko, I. M.; Huang, C.-C.; Kadir, A.; Lee, K. E.; Chua, S. J.; Fitzgerald, E. A. *Semiconductor Science and Technology* **2017**, *32*, 065001.
2. Bravman, J. C.; Sinclair, R. *J. Journal of Electron Microscopy Technique* **1984**, *1*, 53.
3. Reshchikov, M. A.; Morkoç, H. *Journal of Applied Physics* **2005**, *97*, 5.
4. Sun, J.; Ruzsinszky, A.; Perdew, J. P. *Physical Review Letters* **2015**, *115*, 036402.
5. Kresse, G. & Furthmüller, J. Efficient Iterative Schemes for *ab initio* Total-Energy Calculations Using a Plane-Wave Basis Set. *Physical Review B* **54**, 11169 (1996).
6. Kresse, G. & Joubert, D. From Ultrasoft Pseudopotentials to the Projector Augmented-Wave Method. *Physical Review B* **59**, 1758 (1999).
7. Mishra, T. P.; Syaranamual, G. J.; Deng, Z.; Chung, J. Y.; Zhang, L.; Goodman, S. A.; Jones, L.; Bosman, M.; Gradečak, S.; Pennycook, S. J.; et al. *Physical Review Materials* **2021**, *5*, 024605.
8. Chung, J.-Y.; Zhang, L.; Syaranamual, G. J.; Gradečak, S.; Pennycook, S. J.; Bosman, M. *ACS Applied Nano Materials* **2021**, *6*, 14019.
9. Humphreys, C. J.; Griffiths, J. T.; Tang, F.; Oehler, F.; Findlay, S. D.; Zheng, C.; Etheridge, J.; Martin, T. L.; Bagot, P. A. J.; Moody, M. P.; Sutherland, D.; Dawson, P.; Schulz, S.; Zhang, S.; Fu, W. Y.; Zhua, T.; Kappers, M. J.; Oliver, R. A. *Ultramicroscopy* **2017**, *176*, 93.
10. Lee, J. K.; Park, B.; Song, K.; Jung, W. Y.; Tyutyunnikov, D.; Yang, T.; Koch, C. T.; Park, C. G.; van Aken, P. A.; Kim, Y.-M.; Kim, J. K.; Bang J. Chen, L.-Q.; Oh, S. H. *Acta Materialia* **2018**, *145*, 109.
